# Variable Selection in the Regularized Simultaneous Component Analysis Method for Multi-Source Data Integration

**DOI:** 10.1038/s41598-019-54673-2

**Published:** 2019-12-09

**Authors:** Zhengguo Gu, Niek C. de Schipper, Katrijn Van Deun

**Affiliations:** Department of Methodology and Statistics, Tilburg University, Tilburg, 5000 LE The Netherlands

**Keywords:** Chemistry, Statistics

## Abstract

Interdisciplinary research often involves analyzing data obtained from different data sources with respect to the same subjects, objects, or experimental units. For example, global positioning systems (GPS) data have been coupled with travel diary data, resulting in a better understanding of traveling behavior. The GPS data and the travel diary data are very different in nature, and, to analyze the two types of data jointly, one often uses data integration techniques, such as the regularized simultaneous component analysis (regularized SCA) method. Regularized SCA is an extension of the (sparse) principle component analysis model to the cases where at least two data blocks are jointly analyzed, which - in order to reveal the joint and unique sources of variation - heavily relies on proper selection of the set of variables (i.e., component loadings) in the components. Regularized SCA requires a proper variable selection method to either identify the optimal values for tuning parameters or stably select variables. By means of two simulation studies with various noise and sparseness levels in simulated data, we compare six variable selection methods, which are cross-validation (CV) with the “one-standard-error” rule, repeated double CV (rdCV), BIC, Bolasso with CV, stability selection, and index of sparseness (IS) - a lesser known (compared to the first five methods) but computationally efficient method. Results show that IS is the best-performing variable selection method.

## Introduction

As a result of recent technological developments, often data from varying types of sources with respect to the same investigation units are gathered and analyzed jointly, which is referred to as multi-source data integration (also known as multi-block data analysis, linked data analysis, and in a broader sense, data fusion^1^). In health research, joint analysis combining global positioning systems (GPS) data and self-report travel diary data for the same subjects has been shown to be insightful for understanding people’s traveling behavior, purpose, and immediate environment, providing critical information relevant to health research^2^. In metabolomics, to gain a comprehensive picture of the metabolism in a biological system, researchers have conducted joint analysis on the measures obtained from two different instrumental methods, which are Mass-spectrometry (MS) with gas chromatography (GC/MS) and MS with liquid chromatography (LC/MS)^3–5^, on the same samples. Multi-source data integration has also been found useful in epigenetics (e.g., joint analysis on genetic information and environmental factors)^6^, in epidemiology (e.g., joint analysis on behavioral data and genetic data)^7^, and in longitudinal and life course studies (e.g., joint analysis on longitudinal survey data and bio-measures)^8^, to name a few.

A popular multi-source data integration methodology often used in social and behavior research, bioinformatics, and analytical chemistry^9–14^ is the simultaneous component based data integration method (SCA for short). In essence, SCA is an extension of the well-known principal component analysis (PCA) model^15^ to the cases where more than one data block is analyzed. Here, a data block can be, for example, survey data, genetic data, and behavioral data. Under certain constraints imposed on all data blocks, information shared across all data blocks can be extracted and represented by a few components. Thus, by means of dimension reduction, SCA is used to explore and interpret the internal structure that binds all data blocks together. Recent extensions of SCA have greatly improved the flexibility and the usefulness of the method by incorporating regularization such as the Lasso^16^ and the Group Lasso^17^, resulting in the regularized simultaneous component analysis method (regularized SCA for short)^13,18–20^. Regularized SCA reveals not only the information shared across all data blocks, which is often referred to as “the common process” or “the joint sources of variation” in the data, but also the information that is unique to certain but not all data blocks, which is referred to as “the specific process” or “the unique variation” underlying the data. Being able to correctly identify and distinguish the common and specific processes is useful and important. For example, Kuppens, Ceulemans, Timmerman, Diener, and Kim-Prieto^21^ pointed out that, in cross-cultural psychology, researchers were often interested in information that was unique to a certain culture (i.e., the specific process), but unfortunately such unique information was usually buried under a vast volume of common traits shared across all cultures (i.e., the common process) and therefore was difficult to be identified. Regularized SCA can be used to identify such unique information. In addition, regularized SCA can handle high-dimensional datasets and, compared to SCA, not only produces sparse results that are much easier to interpret, but also yields consistent estimates^22^. Such selection of the relevant variables is often needed in practice to hint at what variables to further investigate. As a side note, SCA involves rotating component structure and truncating small loadings to zeros, which may generate misleading results^23^. Regularized SCA, however, does not require the rotation or truncation of results. To explain what regularized SCA can offer, we use an application of the method to a three-block parent-child relationship survey dataset documented by Gu and Van Deun^18^ as an example.

**Figure 1:**
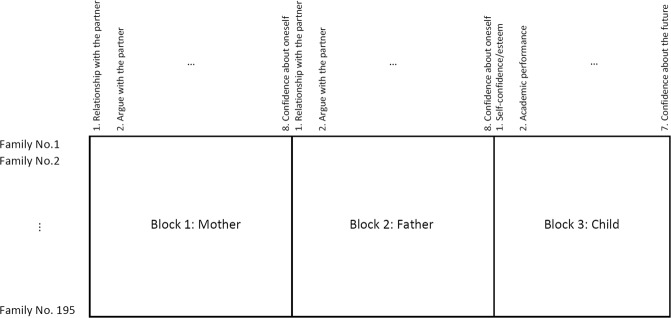
Joint analysis on multi-source data: Using the parent-child relationship survey dataset as an example.

The parent-child relationship survey dataset consists of three data blocks obtained from a large-scale survey collected from 195 families. For details of this dataset, see Gu and Van Deun^18^, and for details of the raw data from which the parent-child relationship survey dataset was retrieved, see Schneider and Waite^24^. The first data block contains 195 mothers’ opinions with respect to 8 items, including (1) relationship with partners, (2) aggressiveness when arguing with the partner, (3) child’s bright future, (4) activities with the child, (5) feelings about parenting, (6) communication with the child, (7) aggressiveness when communicating with the child, and (8) confidence about oneself. The second data block contains 195 fathers’ opinions regarding the same 8 items. The third data block contains 195 children’s ratings on 7 items, including (1) self confidence/esteem, (2) academic performance, (3) social life and extracurricular activities, (4) importance of friendship, (5) self image, (6) happiness, and (7) confidence about the future. Table 1 shows the descriptive statistics of the dataset. The three data blocks can be jointly analyzed because they share the same investigation units – families. In other words, when the three data matrices are placed side by side (see Fig. 1), each row contains the information of the mother, the father, and the child from the same family. The result of regularized SCA (combined with CV for variable selection) applied to this data set is presented in Table 2, which contains an estimated component loading matrix. The individual loadings contained in Table 2 are interpreted in a similar way as the loadings generated in a PCA analysis, but the power of regularized SCA is that it facilitates the interpretation of joint and specific variation at the block level. The table reveals a few important features of regularized SCA. First, the result is sparse, meaning that redundant information is dropped, facilitating easy interpretations. Second, the method reveals joint and specific processes underlying the three data blocks. For example, Component 1 combines information from all three data blocks, capturing the joint process relevant to the parent-child relationship. Components 2, 3, 4, and 5 reveal specific processes that are unique to the parents (i.e., components 2 and 3), unique to the children (i.e., Component 4), and unique to the fathers (i.e., Component 5). To interpret the components, we use Component 3 as an example. This component suggests that for both the mother and the father, their (good) relationship with the partner, (less) aggressiveness when arguing with the partner, and their (high) self-confidence are positively associated among each other.

**Table 1:** Descriptive statistics of the parent-child relationship data, obtained from Gu and Van Deun^18^.

Questionnaire Title	Mean	SD
**Mother**		
Relationship with partners (the higher the score, the more satisfied)	3.58	0.79
Argue with partners (the higher the score, the less violent)	3.65	0.42
Child’s bright future (the higher the score, the stronger the feeling of bright future)	4.49	0.52
Activities with the child (the higher the score, the more activities)	2.40	0.39
Feelings about parenting (the higher the score, the more positive about parenting)	3.33	0.68
Communication with the child (the higher the score, the more communication)	4.16	0.50
Argue (aggressively) with the child (the higher the score, the less aggressive)	3.08	0.45
Confidence about oneself (the higher the score, the more confident)	2.71	0.43
**Father**		
Relationship with partners (the higher the score, the more satisfied)	3.67	0.70
Argue with partners (the higher the score, the less violent)	3.77	0.42
Child’s bright future (the higher the score, the stronger the feeling of bright future)	4.48	0.51
Activities with the child (the higher the score, the more activities)	2.30	0.38
Feelings about parenting (the higher the score, the more positive about parenting)	3.40	0.64
Communication with the child (the higher the score, the more communication)	3.97	0.60
Argue (aggressively) with the child (the higher the score, the less aggressive)	3.18	0.42
Confidence about oneself (the higher the score, the more confident)	2.78	0.47
**Child**		
Self confidence/esteem (the higher the score, the more confident)	2.08	0.46
Academic performance (the higher the score, the better the performance)	6.87	1.32
Social life and extracurricular activities (the higher the score, the more social life)	2.22	0.38
Importance of friendship (the higher the score, the more important friendship is)	3.94	0.61
Self image (the higher the score, the more positive self image is)	2.56	0.52
Happiness (the higher the score, the happier)	2.29	0.44
Confidence about the future (the higher the score, the more confident about the future)	3.94	0.47

**Table 2:** Estimated component loading matrix generated by the regularized SCA method with cross-validation (CV) applied to the parent-child relationship data, obtained from Gu and Van Deun^18^.

	Component 1	Component 2	Component 3	Component 4	Component 5
**Mother**					
Relationship with partners	0	0	11.92	0	0
Argue with partners	−5.53	0	5.88	0	0
Childs bright future	−8.83	0	0	0	0
Activities with children	−4.65	−9.02	0	0	0
Feeling about parenting	−9.02	0	0	0	0
Communation with children	−9.20	0	0	0	0
Argue with children	−8.78	0	0	0	0
Confidence about oneself	−6.66	0	7.26	0	0
**Father**					
Relationship with partners	0	0	11.80	0	0
Argue with partners	0	0	5.26	0	−9.17
Childs bright future	−3.39	0	0	0	−5.76
Activities with children	0	−11.56	0	0	0
Feeling about parenting	−4.04	0	0	0	−6.94
Communation with children	0	−8.17	0	0	0
Argue with children	−4.98	0	0	0	−9.88
Confidence about oneself	0	0	5.60	0	−8.19
**Child**					
Self confidence/esteem	−5.82	0	0	8.66	0
Academic performance	0	0	0	7.08	0
Social life and extracurricular	0	0	0	4.10	0
Importance of friendship	0	0	0	9.60	0
Self Image	0	0	0	10.36	0
Happiness	0	0	0	9.55	0
Confidence about the future	0	0	0	7.48	0

Note that we are interested in the associations among items within a component, and the associations are indicated by the signs of the loadings. Take Component 2 for example. The three non-zero loadings have the same sign (in this case “−” sign), meaning that mother’s “activities with children”, father’s “activities with children”, and father’s “communication with children” are positively associated with each other. Two loadings having opposite signs indicates a negative association between the two items. We remind the reader that, when interpreting the loadings and the associations among them, one should also take into account how the items are scored (see Table 1). For example, a higher score on “relationship with parters” indicates a *more* satisfied relationship. A higher score on “argue with partners” indicates a *less* violent relationship.

The parent-child relationship example shows that regularized SCA can be a powerful tool for jointly exploring multiple data sources and discovering interesting internal structures shared among data sources or unique to some but not all data sources. However, to realize its full potential, regularized SCA requires a proper variable selection method for component loadings to ensure that the right structure (i.e., whether components are common or unique) and the right level of sparseness are imposed. Currently, CV with “one-standard-error” rule and stability selection^25^ have been used together with regularized SCA^19,20^. As far as we know, no research has been conducted on the performance of the two variable selection methods: We do not know whether the two methods indeed correctly select important variables (i.e., non-zero component loadings), and if they do, which variable selection method performs better. CV and stability selection are not the only methods for regularized SCA. Other variable selection methods, including information-criterion-based indices and bootstrapping methods, have been proposed for regularized models, such as sparse PCA and regularized regression analysis, but they have not been used for regularized SCA.

In this study, to identify a suitable variable selection method for regularized SCA, we examined the performance of six methods, including CV with “one-standard-error” rule^26^, stability selection^25^, repeated double cross-validation (rdCV)^27^, Index of Sparseness (IS)^28–30^, Bolasso with CV^31–33^, and a BIC criterion^34,35^. We chose CV with the “one-standard-error” rule, rdCV, IS, and Bolasso, because they had been used successfully in various applications of sparse PCA methods, including early recognition and disease prediction^36^, schizophrenia research^37^, epidemics^38^, cardiac research^39^, environmental research^40^, and psychometrics^41^. We included stability selection because of its popularity in the statistical literature and because it has been used for regularized SCA. We included the BIC criteria by Croux, Filzmoser and Fritz^34^ and by Guo, James, Levina, Michailidis, and Zhu^35^ and IS because of their computational efficiency. In addition, we provided an adjusted algorithm of stability selection specifically designed for regularized SCA, and we explained how to use rdCV, IS, Bolasso with CV and the BIC criterion in regularized SCA.

## Results

### Simulation studies

#### Data generation

We conducted two simulation studies. In the first simulation study, we evaluated the performance of the variable selection methods when two data blocks were integrated. We considered high dimensional data blocks (i.e., the number of persons smaller than that of variables) and also typical data blocks seen in social sciences (i.e., the number of persons larger than that of variables). The second simulation study extended the first one by integrating four data blocks rather than two data blocks. Both simulation studies followed the same simulation design, and therefore, in the remainder of the section, we outline the design of the first simulation study in details and mention the second simulation study when necessary.

In the first simulation study, the data were generated in five steps.

Step 1: Two data matrices, denoted by **X**_1_ and **X**_2_, were generated. Here we considered three situations:

1 $$(1)\,{{\bf{X}}}_{1}=\{{x}_{ij}\}\in { {\mathcal R} }^{20\times 40}\,{\rm{and}}\,{{\bf{X}}}_{2}=\{{x}_{ij}\}\in { {\mathcal R} }^{20\times 10},$$

2 $$(2)\,{{\bf{X}}}_{1}=\{{x}_{ij}\}\in { {\mathcal R} }^{20\times 120}\,{\rm{and}}\,{{\bf{X}}}_{2}=\{{x}_{ij}\}\in { {\mathcal R} }^{20\times 30},$$

and

3 $$(3)\,{{\bf{X}}}_{1}=\{{x}_{ij}\}\in { {\mathcal R} }^{80\times 40}\,{\rm{and}}\,{{\bf{X}}}_{2}=\{{x}_{ij}\}\in { {\mathcal R} }^{80\times 10},$$

where, for all three situations, ${x}_{ij}\sim i.i.d.N(0,1)$. The choice of how to generate initial structures in this step has little influence on the final results as it only contributes to the true model part; other choices could also have been made, for example using an autoregressive structure on the covariance matrices. Then, the concatenated data matrix with respect to rows, denoted by ${\tilde{{\bf{X}}}}_{C}=[{{\bf{X}}}_{1},{{\bf{X}}}_{2}]$, was of dimension 20 × 50, 20 × 150, and 80 × 50, respectively. In the following, we use the first situation (i.e., Eq. 1) as an example to explain the remaining steps.

Step 2: Using singular value decomposition (SVD), we decomposed ${\tilde{{\bf{X}}}}_{C}$ into **U**$\Sigma$**V**. We defined the “true” component score matrix, denoted by **T**^*true*^, as the matrix containing the three left singular vectors in **U** corresponding to the three largest singular values. Let $\tilde{\Sigma }$ denote the diagonal matrix containing the three largest singular values, and let $\tilde{{\bf{V}}}$ denote the matrix containing the three right singular vectors corresponding to the three largest singular values. Then, the non-sparse component loading matrix, denoted by ${\tilde{{\bf{P}}}}_{C}$, was ${\tilde{{\bf{P}}}}_{C}=\tilde{{\bf{V}}}\tilde{\Sigma }$.

Step 3: Notice that ${\tilde{{\bf{P}}}}_{C}$ is a 50 × 3 matrix. Let ${\tilde{{\bf{P}}}}_{1}\equiv [{{\bf{p}}}_{1}^{1},{{\bf{p}}}_{2}^{1},{{\bf{p}}}_{3}^{1}]\in { {\mathcal R} }^{40\times 3}$ denote the component loading matrix corresponding to the first block. Let ${\tilde{{\bf{P}}}}_{2}\equiv [{{\bf{p}}}_{1}^{2},{{\bf{p}}}_{2}^{2},{{\bf{p}}}_{3}^{2}]\in { {\mathcal R} }^{10\times 3}$ denote the component loading matrix corresponding to the second block. Thus, $${\tilde{{\bf{P}}}}_{C}\equiv [\begin{array}{c}{\tilde{{\bf{P}}}}_{1}\\ {\tilde{{\bf{P}}}}_{2}\end{array}]$$. We assumed that the first component of ${\tilde{{\bf{P}}}}_{C}$ was the common component, representing the common process across both data blocks, and we assumed that remaining two components were distinctive components, representing unique processes, so that ${{\bf{p}}}_{2}^{1}$ in ${\tilde{{\bf{P}}}}_{1}$ and ${{\bf{p}}}_{3}^{2}$ in ${\tilde{{\bf{P}}}}_{2}$ were replaced with **0**. As a result, ${\tilde{{\bf{P}}}}_{C}$ became $$[\begin{array}{ccc}{{\bf{p}}}_{1}^{1} & {\bf{0}} & {{\bf{p}}}_{3}^{1}\\ {{\bf{p}}}_{1}^{2} & {{\bf{p}}}_{2}^{2} & {\bf{0}}\end{array}]$$.

Step 4: We replaced some loadings in ${{\bf{p}}}_{1}^{1}$, ${{\bf{p}}}_{1}^{2}$, ${{\bf{p}}}_{2}^{2}$, and ${{\bf{p}}}_{3}^{1}$ with zeros to make ${{\bf{p}}}_{1}^{1}$, ${{\bf{p}}}_{1}^{2}$, ${{\bf{p}}}_{2}^{2}$, and ${{\bf{p}}}_{3}^{1}$ sparse, and we considered two situations: 30% and 50% of the loadings in ${{\bf{p}}}_{1}^{1}$, ${{\bf{p}}}_{1}^{2}$, ${{\bf{p}}}_{2}^{2}$, and ${{\bf{p}}}_{3}^{1}$ were replaced with zeros. Let ${{\bf{P}}}_{C}^{true}$ denote the concatenated component loading matrix after the sparseness was introduced to $$[\begin{array}{ccc}{{\bf{p}}}_{1}^{1} & {\bf{0}} & {{\bf{p}}}_{3}^{1}\\ {{\bf{p}}}_{1}^{2} & {{\bf{p}}}_{2}^{2} & {\bf{0}}\end{array}]$$. Note that for notational convenience we used the same symbols for the sparsified loading vectors as previously.

Step 5: We computed ${{\bf{X}}}_{C}^{true}={{\bf{T}}}^{true}{({{\bf{P}}}_{C}^{true})}^{T}$, and added a noise matrix, denoted by **E**, to ${{\bf{X}}}_{C}^{true}$ to generate the final simulated dataset, denoted by ${{\bf{X}}}_{C}^{generated}$, so that ${{\bf{X}}}_{C}^{generated}={{\bf{X}}}_{C}^{true}+\alpha {\bf{E}}$, where the scalar *α* is a scaling factor. The cells in **E** were generated from $N(0,1)$. Note that an implicit assumption of PCA and also SCA is independent and identically distributed noise; other types of noise structure may affect the results. By adjusting *α*, we were able to control the proportion of noise variance in ${{\bf{X}}}_{C}^{generated}$. We considered two noise levels: 0.5% and 30% of variance in ${{\bf{X}}}_{C}^{generated}$ were attributable to noise.

In summary, the first simulation study included the following design factors:

- Three situations of **X**_1_ and **X**_2_ (i.e., Eqs. 1, 2 and 3).
- Two sparseness levels in ${{\bf{p}}}_{1}^{1}$, ${{\bf{p}}}_{1}^{2}$, ${{\bf{p}}}_{2}^{2}$, and ${{\bf{p}}}_{3}^{1}$: 30% and 50%.
- Two noise levels: 0.5%, and 30%.
  The design factors were fully crossed, resulting in $3\times 2\times 2=12$ design cells. In each design cell, we simulated 20 datasets following the above five steps, and therefore in total 240 datasets were simulated. Then, for each dataset, we conducted the regularized SCA analysis and compared the results generated by the model selection methods, which are CV with “one-standard-error” rule, rdCV, BIC, IS, Bolasso with CV, and stability selection.
  The design of the second simulation study also involved five steps similar to the first simulation, but we made the following changes. In Step 1 of the second simulation study, we considered only one situation:
  4 $$\begin{array}{rcl}{{\bf{X}}}_{1} & = & \{{x}_{ij}\}\in { {\mathcal R} }^{20\times 120},\\ {{\bf{X}}}_{2} & = & \{{x}_{ij}\}\in { {\mathcal R} }^{20\times 30},\\ {{\bf{X}}}_{3} & = & \{{x}_{ij}\}\in { {\mathcal R} }^{20\times 40},\,{\rm{and}}\\ {{\bf{X}}}_{4} & = & \{{x}_{ij}\}\in { {\mathcal R} }^{20\times 10},\end{array}$$
  where ${x}_{ij}\sim i.i.d.N(0,1)$. In Step 3, we inserted **0** in ${\tilde{{\bf{P}}}}_{C}$ such at
  5 $${\tilde{{\bf{P}}}}_{C}=[\begin{array}{ccc}{{\bf{p}}}_{1}^{1} & {{\bf{p}}}_{2}^{1} & {\bf{0}}\\ {{\bf{p}}}_{1}^{2} & {{\bf{p}}}_{2}^{2} & {{\bf{p}}}_{3}^{2}\\ {{\bf{p}}}_{1}^{3} & {\bf{0}} & {{\bf{p}}}_{3}^{3}\\ {{\bf{p}}}_{1}^{4} & {\bf{0}} & {\bf{0}}\end{array}].$$
  In summary, the second simulation study included the following two design factors:
- Two sparseness levels in ${{\bf{p}}}_{1}^{1}$, ${{\bf{p}}}_{2}^{1}$, ${{\bf{p}}}_{1}^{2}$, ${{\bf{p}}}_{2}^{2}$, ${{\bf{p}}}_{3}^{2}$, ${{\bf{p}}}_{1}^{3}$, ${{\bf{p}}}_{3}^{3}$, and ${{\bf{p}}}_{1}^{4}$: 30% and 50%.
- Two noise levels: 0.5%, and 30%.

The design factors were fully crossed, resulting in $2\times 2=4$ design cells. In each design cell, we simulated 20 datasets following the above five steps, and therefore in total 80 datasets were simulated.

#### Performance measures

To compare the variable selection methods, we used two types of performance measures. The first type concerned the component loading matrix, and the second type concerned the component score matrix. The first type consisted of three performance measures. Let ${\hat{{\bf{P}}}}_{C}$ denote the estimated concatenated component loading matrix. The first performance measure, denoted by *PL*, was the proportion of non-zero and zero loadings correctly identified in ${\hat{{\bf{P}}}}_{C}$ compared to ${{\bf{P}}}_{C}^{true}$:

6 $$PL=\tfrac{{\rm{number}}\,{\rm{of}}\,{\rm{correctly}}\,{\rm{selected}}\,{\rm{non}}-{\rm{zero}}\,{\rm{loadings}}+{\rm{number}}\,{\rm{of}}\,{\rm{correctly}}\,{\rm{identified}}\,{\rm{zero}}\,{\rm{loadings}}}{{\rm{total}}\,{\rm{number}}\,{\rm{of}}\,{\rm{loadings}}\,{\rm{in}}\,{{\bf{P}}}_{C}^{true}}\mathrm{}.$$

Notice that $PL\in [0,1]$. Intuitively, for regularized SCA, the best model selection method should be the one that generating the highest *PL* among the methods. In addition to *PL*, we also used *PL*_non-0 loadings_, defined as

7 $$P{L}_{{\rm{non}}-0{\rm{loadings}}}=\tfrac{{\rm{number}}\,{\rm{of}}\,{\rm{correctly}}\,{\rm{selected}}\,{\rm{non}}-{\rm{zero}}\,{\rm{loadings}}}{{\rm{total}}\,{\rm{number}}\,{\rm{of}}\,{\rm{non}}-{\rm{zero}}\,{\rm{loadings}}\,{\rm{in}}\,{{\bf{P}}}_{C}^{true}},$$

and *PL*_0 loadings_, defined as

8 $$P{L}_{0{\rm{loadings}}}=\tfrac{{\rm{number}}\,{\rm{of}}\,{\rm{correctly}}\,{\rm{identified}}\,{\rm{zero}}\,{\rm{loadings}}}{{\rm{total}}\,{\rm{number}}\,{\rm{of}}\,{\rm{zero}}\,{\rm{loadings}}\,{\rm{in}}\,{{\bf{P}}}_{C}^{true}}\mathrm{}.$$

We used *PL*_non-0 loadings_ to evaluate how well a model selection method assisted correctly retaining non-zero loadings and used *PL*_0 loadings_ to evaluate how well a model selection method assisted correctly identifying zero loadings.

In this study, we focused on the component loading matrix, and we used the variable selection methods to help us identify non-zero and zero loadings, but the component score matrix was also important. Ideally, we would prefer an estimated component score matrix as close as possible to the true component score matrix. Therefore, the second type of performance measure evaluated the degree of similarity between **T**^*true*^ and the estimated component score matrix $\hat{{\bf{T}}}$, quantified by Tucker congruence $\phi$^42^

9 $$\phi =\frac{{\rm{vec}}{({{\bf{T}}}^{true})}^{T}{\rm{vec}}(\hat{{\bf{T}}})}{\sqrt{({\rm{vec}}{({{\bf{T}}}^{true})}^{T}{\rm{vec}}({{\bf{T}}}^{true}))({\rm{vec}}{(\hat{{\bf{T}}})}^{T}{\rm{vec}}(\hat{{\bf{T}}}))}}.$$

Notice that $\phi \in [\,-\,1,1]$. Ideally, a good model selection method for regularized SCA is the one that makes $\phi$ close to 1.

#### Results

We used the R package RegularizedSCA (version 0.5.5)^20^ to estimate the regularized SCA model; the R script for replicating the study is included in the supplementary material. All columns in the simulated datasets were mean-centered and scaled to norm one. We used the Group Lasso penalty to identify component structure (i.e., common/distinctive components) and used the Lasso penalty to impose sparseness within a component. For details, please see the Methods section.

**Figure 2:**
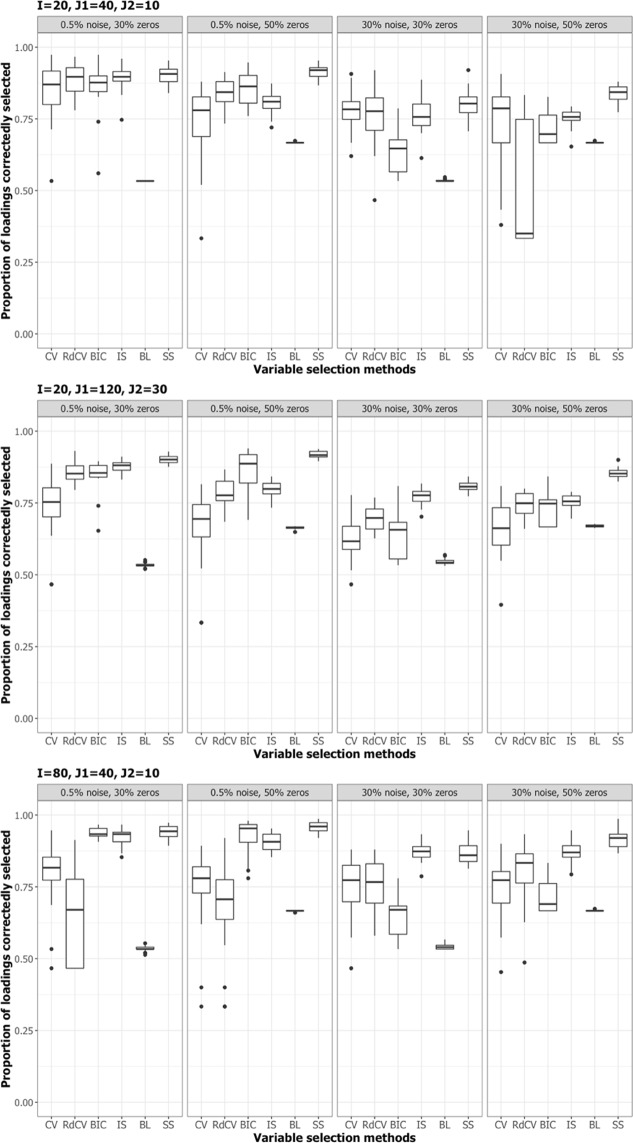
Integration of two blocks: Proportion of non-zero and zero loadings in ${\hat{{\bf{P}}}}_{C}$ correctly identified (i.e., *PL*). The upper, middle, and bottom panels correspond to Eqs. 1, 2 and 3, respectively. BL stands for BoLasso with CV. SS stands for stability selection.

**Figure 3:**
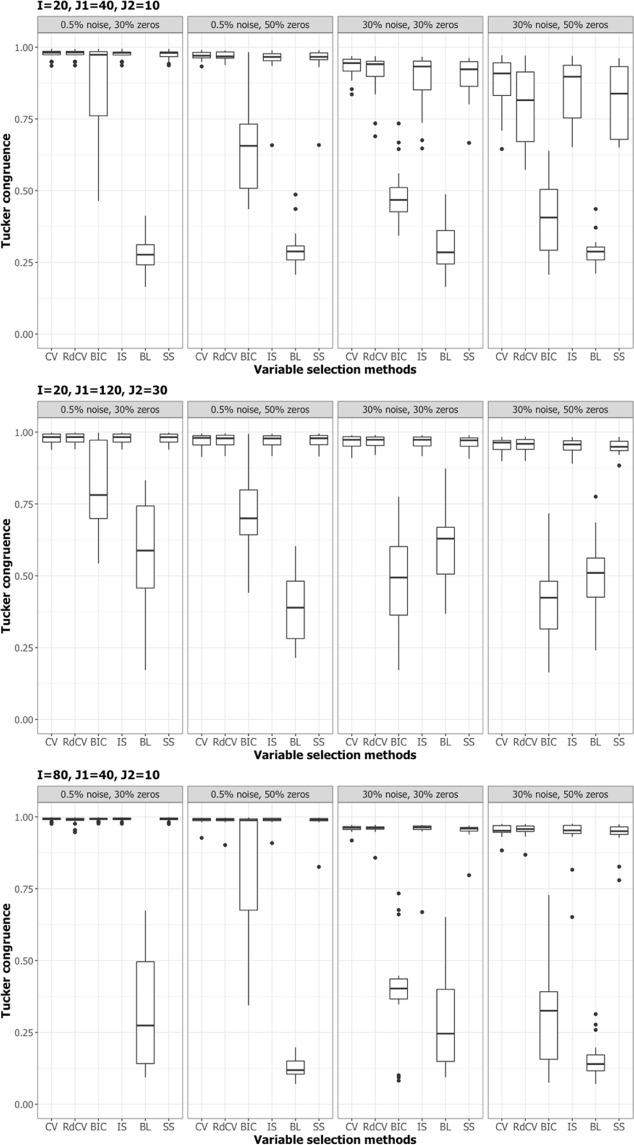
Integration of two blocks: Tucker congruences between $\hat{{\bf{T}}}$ and **T**. The upper, middle, and bottom panels correspond to Eqs. 1, 2 and 3, respectively. BL stands for BoLasso with CV. SS stands for stability selection.

**Figure 4:**
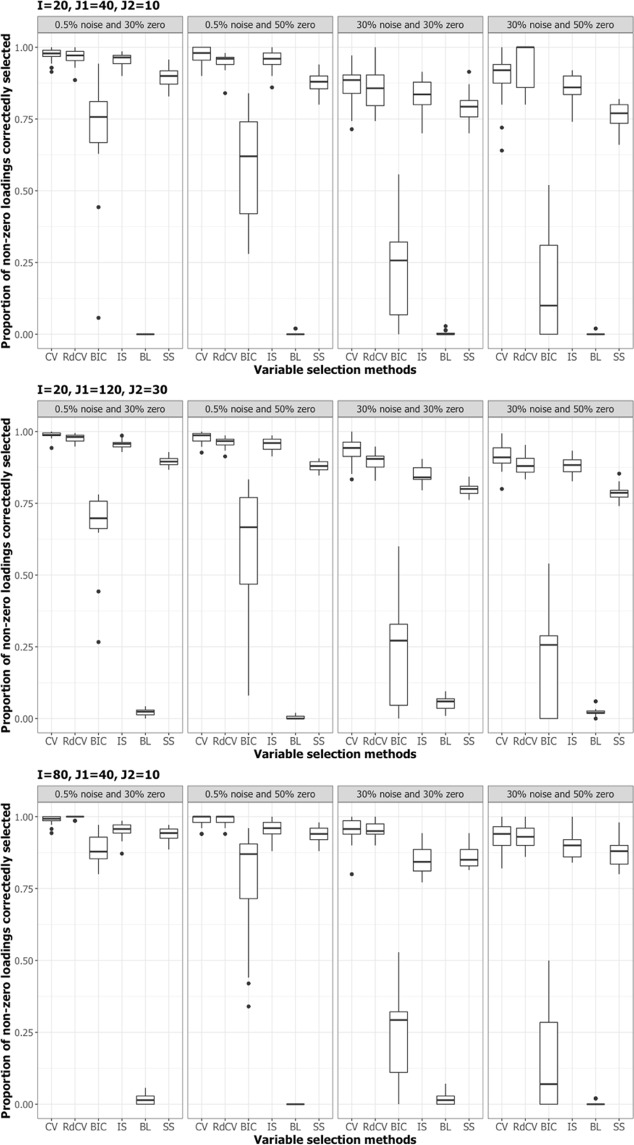
Integration of two blocks: Proportion of non-zero loadings in ${\hat{{\bf{P}}}}_{C}$ correctly selected (i.e., *PL*_non-0 loadings_). BL stands for BoLasso with CV. SS stands for stability selection.

**Figure 5:**
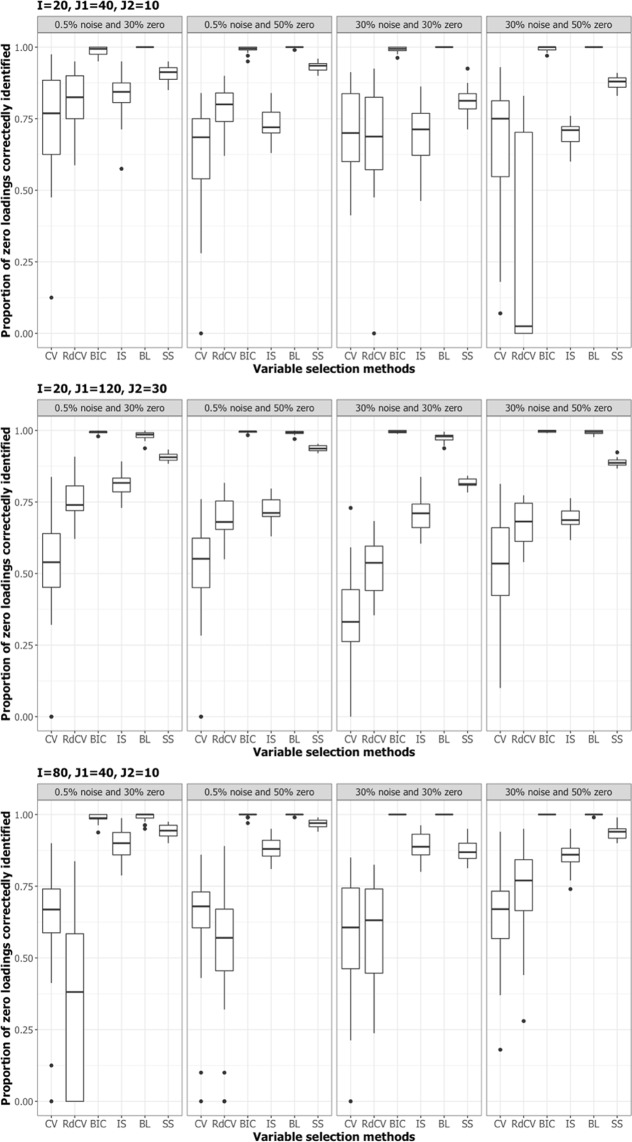
Integration of two blocks: Proportion of zero loadings in ${\hat{{\bf{P}}}}_{C}$ correctly identified (i.e., *PL*_0 loadings_). BL stands for BoLasso with CV. SS stands for stability selection.

Figures 2, 3, 4 and 5 summarize the results of the first simulation, where two data blocks were integrated. Specifically, Figs. 2, 4 and 5, by means of boxplots, present the performance measures *PL* (Eq. 6), *PL*_non-0 loadings_ (Eq. 7), and *PL*_0 loadings_ (Eq. 8), respectively. Figure 3 presents the boxplots of Tucker congruence measures. For each figure, the upper, middel, and bottom panels correspond to the first, second, and third situations of **X**_1_ and **X**_2_ (i.e., Eqs. 1, 2 and 3), respectively. The reader may notice that most methods (except for BIC and Bolasso) did not differ much in Tucker congruence, and therefore, we focus on discussing *PL*, *PL*_non-0 loadings_, and *PL*_0 loadings_ and mention Tucker conguence only when necessary.

**Figure 6:**
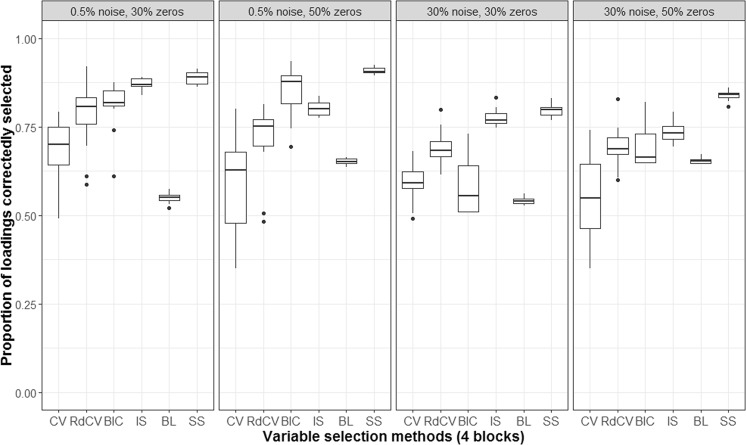
Integration of four blocks: Proportion of non-zero and zero loadings in ${\hat{{\bf{P}}}}_{C}$ correctly identified (i.e., *PL*). BL stands for BoLasso with CV. SS stands for stability selection.

**Figure 7:**
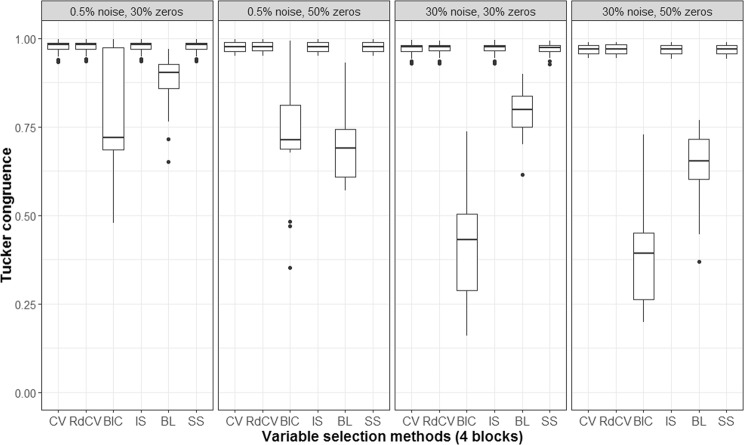
Integration of four blocks: Tucker congruences between $\hat{{\bf{T}}}$ and **T**. BL stands for BoLasso with CV. SS stands for stability selection.

**Figure 8:**
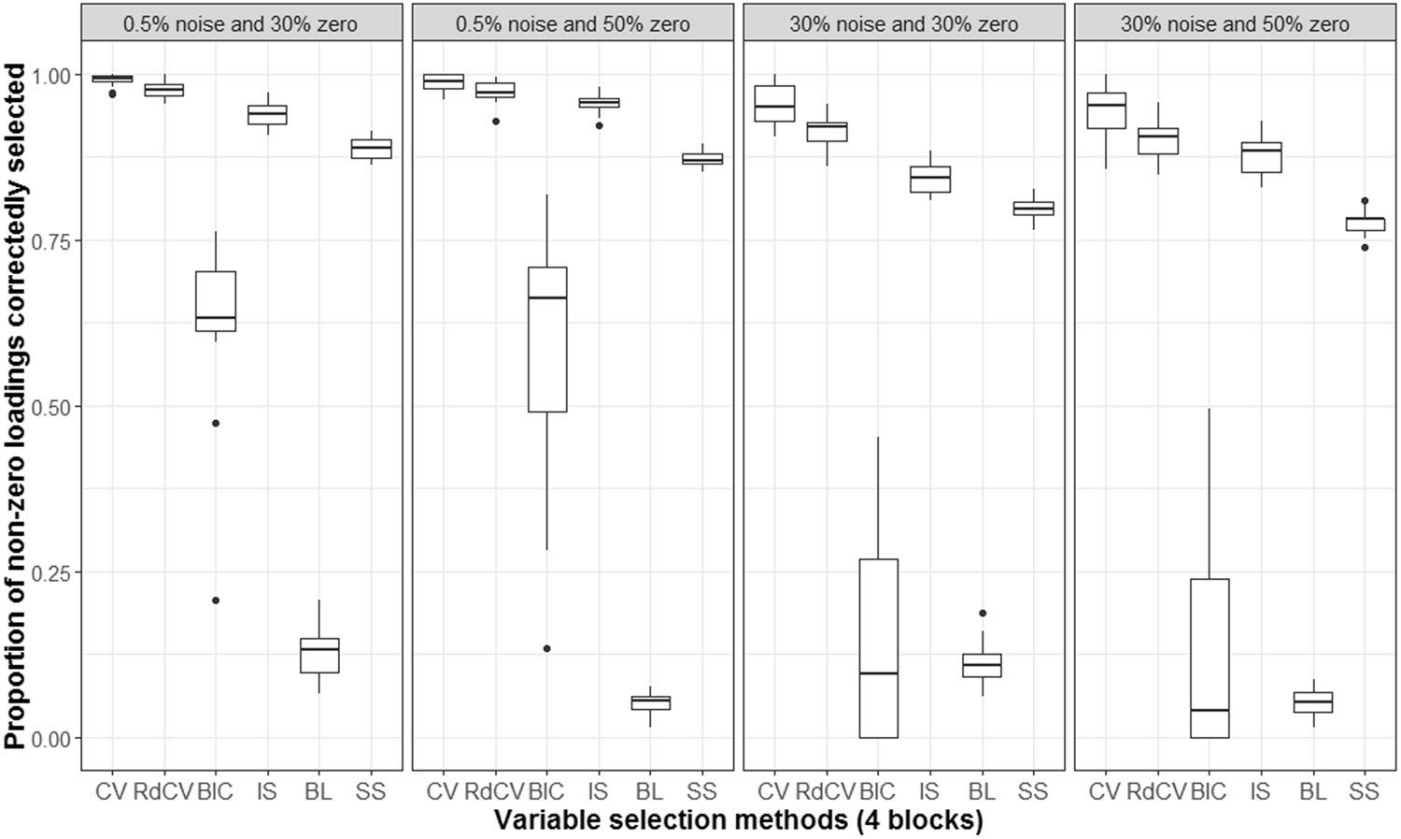
Integration of four blocks: Proportion of non-zero loadings in ${\hat{{\bf{P}}}}_{C}$ correctly selected (i.e., *PL*_non-0 loadings_). BL stands for BoLasso with CV. SS stands for stability selection.

**Figure 9:**
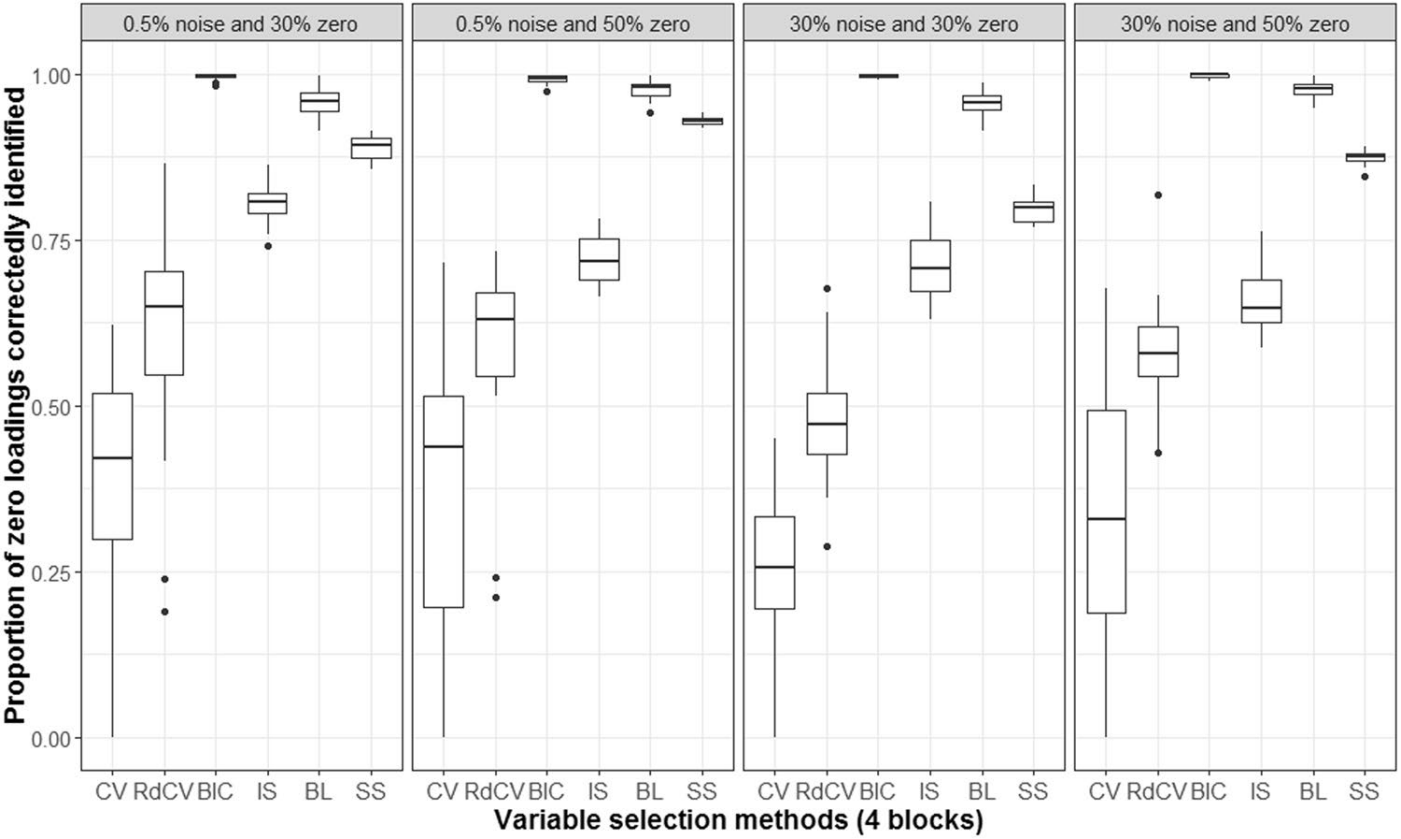
Integration of four blocks: Proportion of zero loadings in ${\hat{{\bf{P}}}}_{C}$ correctly identified (i.e., *PL*_0 loadings_). BL stands for BoLasso with CV. SS stands for stability selection.

Based on the figures, we concluded the following. First, CV with “one-standard-error” rule and rdCV did not outperform the other methods in most cases in terms of correctly identifying non-zero and zero loadings (see Fig. 2). Figures 4 and 5 show that the two methods tended to retain more non-zero loadings than needed, resulting in high *PL*_non-0 loadings_ but low *PL*_0 loadings_, which is a known feature of CV-based methods^43^. Second, stability selection was the best-performing method in terms of *PL*. However, as we have explained in the Methods section, in order for the method to work in the simulation, we assumed that the correct number of non-zero loadings was known a priori, which is unrealistic in practice. Third, IS was the second best-performing method (Fig. 2), witnessed by a balanced, high *PL*_non-0 loadings_ (Fig. 4) and high *PL*_0 loadings_ (Fig. 5). Fourth, BIC performed worse than the other methods (except for Bolasso) when the noise level was high (i.e., 30%). Figures 4 and 5 suggest that BIC consistently favored very sparse results, resulting in very high *PL*_0 loadings_ but low *PL*_non-0 loadings_, which in turn lead to low Tucker congruence values (Fig. 3). Finally, Bolasso performed the worst among all the methods in terms of *PL* and Tucker congruence. This is primarily because the algorithm is very strict: A loading was identified as a non-zero loading only if the loading was estimated to be different from zero in all 50 repetitions (see the Methods section). As a result, the algorithm generated an estimated loading matrix with too many zeros - that is, very high *PL*_0 loadings_ in Fig. 5 and very low *PL*_non-0 loadings_ in Fig. 4. Figures 6, 7, 8 and 9 present the results of the second simulation study, where four data blocks were integrated. It may be noted that the four figures are very similar to the Figs. 2, 3, 4 and 5, and therefore, similar conclusions can be made for the second simulation study. For the sake of simplicity, we do not discuss the Figs. 6, 7, 8, and 9.

Based on the two simulation studies, we conclude that, in practice, IS is the best-performing variable selection method for regularized SCA. In addition, more research is needed to improve the stability selection algorithm for regularized SCA so that it will no longer rely on the unrealistic assumption that the correct number of total non-zero loading is known a priori.

### Empirical examples

In this section, we present three empirical applications of regularized SCA combined with IS for variable selection. We used the first two empirical examples to explain to the reader how to interpret the estimated component loading matrix generated by regularized SCA together with IS in applied research. The third empirical example is the parent-child relationship data discussed in the Introduction section. We reanalyzed the data by using IS and compared the results with Table 2. We remind the reader that, to evaluate and to interpret the results generated by regularzed SCA, one typically resorts to both the estimated component loading matrix and the estimated component score matrix. In this article, because we focus on variable selection in the component loading matrix, we refrain from discussing the interpretation of the estimated component score matrix in the remainder of this section. Furthemore, for detailed explanation on the use of regularized SCA and the interpretation of the results, we refer to Gu and Van Deun^18^.

We used the following setup for IS: 50 Lasso tuning parameter values (equally spaced ranging from 0.0000001 to the smallest value making the entire estimated component loading matrix a zero matrix), and 50 Group Lasso tuning parameter values (equally spaced ranging from 0.0000001 to the smallest value making the entire estimated component loading matrix a zero matrix). All columns in the empirical datasets were mean-centered and scaled to norm one before the regularized SCA analysis was performed.

#### Joint analysis of the Herring data

In food science, researchers are often interested in the chemical/physical characteristics and the sensory characteristics of a certain food item and analyze the characteristics jointly. An example is the Herring data obtained from a ripening experiment^44,45^. In this article, we used part of the original Herring data^20^, consisting of two datablocks. The first block contained the physical and chemical changes, including pHB, ProteinM, ProteinB, Water, AshM, Fat, TCAIndexM, TCAIndexB, TCAM, and TCAB, of 21 salted herring samples. The meaning of the labels of the physical and chemical changes can be found at http://www.models.life.ku.dk/Ripening_of_Herring. The second block contained the sensory data, including features such as ripened, rawness, malt, stockfish smell, sweetness, salty, spice, softness, toughness, and watery, of the same 21 samples. An interesting research question is whether certain physical and chemical changes are associated with certain sensory characteristics of the herrings. It may be noted that, in this article, we do not discuss how to identify the number of components *R* (see the Methods section), and for this topic, we refer to Gu and Van Deun^18^. A previous study^18^ suggested that, for the Herring data, the reasonable number of components *R* was 4. Therefore, we performed the regularized SCA analysis with IS and $R=4$, and the estimated component loading matrix is presented in Table 3. The table suggests that, for each component, not all variables were important. For example, for Component 1, variables pHB, Water, and AshM from the block of “physical and chemical changes” and variables Ripened, Rawness, Stockfish smell, Sweetness, and Spice from the “sensory” block were important and therefore their loadings were different from zero. To interpret the associations among the variables of Component 1, we primarily look at the signs of the non-zero loadings. For example, for Component 1, variables pHB, Water, Rawness, Sweetness, and Spice were negatively associated with variables AshM, Ripened, Stockfish smell. The remaining three components can be interpreted in the same way.

**Table 3:** The Herring data: Estimated component loading matrix generated by using regularized SCA with IS.

	Component 1	Component 2	Component 3	Component 4
**Physical and chemical changes**				
pHB	2.98	−1.13	0	2.19
ProteinM	0	2.85	0	−2.97
ProteinB	0	−4.04	−1.35	0.87
Water	0.78	−0.78	0	4.27
AshM	−3.67	0	0	2.13
Fat	0	0	0	−4.26
TCAIndexM	0	−4.17	0	0
TCAIndexB	0	0	1.46	−3.97
TCAM	0	−4.09	0	0
TCAB	0	−4.18	−0.73	−0.93
**Sensory**				
Ripened	−1.68	−4.02	0	−0.69
Rawness	1.13	2.90	2.46	0
Malt	0	−4.14	0.95	0
Stockfish smell	−3.84	−0.99	0	−1.58
Sweetness	1.26	−3.45	0	1.21
Salty	0	0	−4.11	0
Spice	1.23	−1.16	−2.68	0.90
Softness	0	−4.34	0	0
Toughness	0	−4.32	0	0
Watery	0	−4.05	0	1.09

#### Joint analysis of metabolomics data

**Figure 10:**
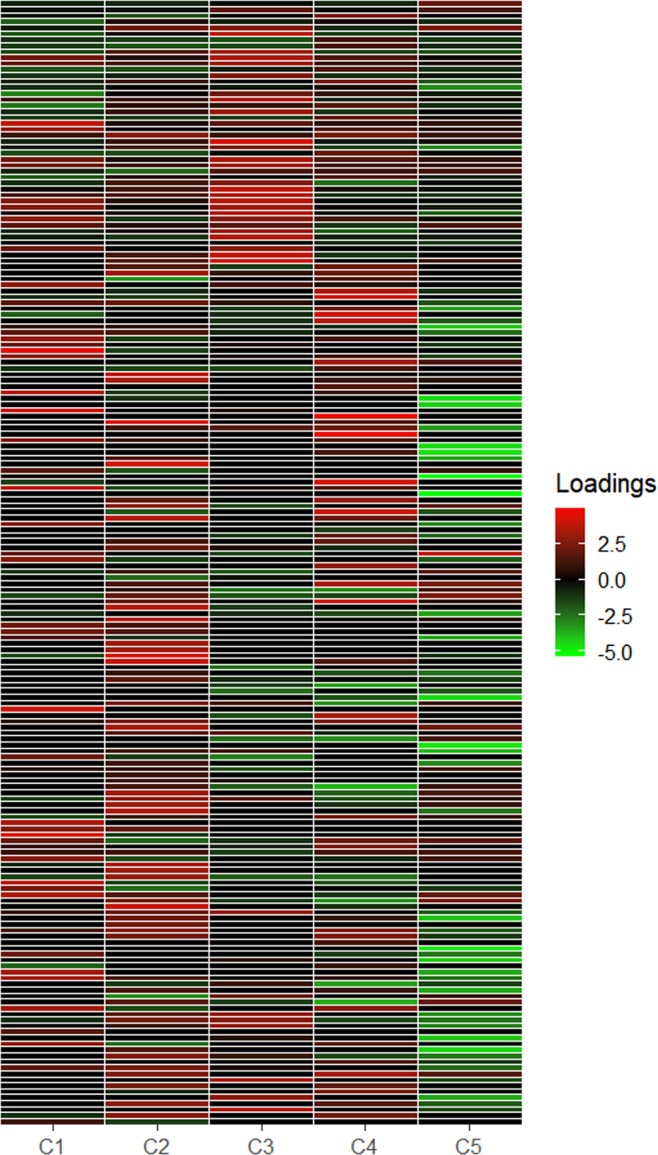
Joint analysis of metabolomics data: The heatmap for the estimated component loading matrix generated by using IS.

In metabolomics, researchers often use multiple instrumental methods to measure as many metabolites as possible and perform joint analyses by combining the measures on the same metabolites gathered from different instrumental methods^5^. The dataset used in this article contained measures of 28 samples of *Escherichia coli* (*E*. *coli*) obtained from using two measurement methods, which were mass spectrometry with gas chromatograph (GC/MS) and mass spectrometry with liquid chromatography (LC/MS)^3,4^. The dataset contained a block of GC/MS data with 144 metabolites and a block of LC/MS data with 44 metabolites. For a detailed description of the dataset, including the experimental design and conditions for obtaining the measures, we refer to Smilde, Van der Werf, Bijlsma, Van der Werff-van der Vat, and Jellema^5^. A previous study^19^ suggested that the appropriate number of components *R* was five. We thus performed the regularized SCA analysis with IS and $R=5$. It may be noted that, in this example, because of the large number of variables, a table of estimated component loading matrix such like Table 3 usually is not practical. Instead, researchers typically use a heatmap so as to get some impression about the sparseness of the loading matrix. Figure 10 presents such a heatmap for the estimated component loading matrix. We found that many loadings in Fig. 10 were very close or equal to zero. As a side note, for this study, researchers typically focus on interpreting the estimated component score matrix instead of the estimated component loading matrix (see, e.g., Van Deun, Wilderjans, van den Berg, Antoniadis, and Van Mechelen^46^).

#### Re-analysis of the parent-child relationship survey data

Table 4 presents the estimated component loading matrix obtained by using IS. The orders of the components were adjusted by using Tucker congruence so that the components in Table 4 are comparable to the components in Table 2 which were generated by using CV^18^. The two estimated component loading matrices in Tables 4 and 2 are comparable, and the conclusions based on the two tables are almost the same. For example, for Component 1 of both tables, the last 7 variables from the “Mother” block were positively associated with the variables “child’s bright future”, “feeling about parenting”, “argue with children” from the “Father” block and were also positively associated with the variable “self-confidence/esteem” from the “Child” block.

**Table 4:** The parent-child relationship data: Estimated component loading matrix generated by using regularized SCA with IS.

	Component 1	Component 2	Component 3	Component 4	Component 5
**Mother**					
Relationship with partners	0	0	12.05	0	0
Argue with partners	−5.42	0	5.74	0	0
Childs bright future	−8.88	0	0	0	0
Activities with children	−4.09	−8.71	0	0	0
Feeling about parenting	−8.85	0	2.80	0	0
Communation with children	−8.77	−3.81	0	0	0
Argue with children	−9.07	0	0	0	0
Confidence about oneself	−6.45	0	7.35	0	0
**Father**					
Relationship with partners	0	0	11.85	0	0
Argue with partners	0	0	5.12	0	−9.27
Childs bright future	−3.53	0	0	0	−5.63
Activities with children	0	−10.87	0	0	0
Feeling about parenting	−4.17	0	0	0	−6.84
Communation with children	0	−8.71	0	0	0
Argue with children	−5.07	0	0	0	−9.83
Confidence about oneself	0	0	5.51	0	−8.29
**Child**					
Self confidence/esteem	−5.88	0	0	8.65	0
Academic performance	0	0	0	7.12	0
Social life and extracurricular	0	0	0	4.03	0
Importance of friendship	0	0	0	9.57	0
Self Image	0	0	0	10.44	0
Happiness	0	0	0	9.64	0
Confidence about the future	0	−4.72	0	7.19	0

Please be noted that the signs of components 1, 2, and 5 were manually changed from positive to negative. The signs of Component 3 were manually changed from negative to positive. Due to the invariance of signs of regularized SCA, changing signs do not influence the interpretation of loadings. Therefore, we changed the signs to make it easier for the reader to compare the table with Table 2.

## Discussion

In this article, we examined six variable selection methods suitable for regularized SCA. The popular CV-based variable selection methods, including CV with “one-standard-error” rule and rdCV, did not outperform other methods. This result may be surprising to many researchers, especially considering that CV seems to be the standard practice when it comes to variable selection. The poor recovery rate of component loadings by using the CV-based methods in the simulations showed that the CV-based methods retained more loadings than needed. Stability selection is a promising method, but at this moment we do not know how to identify an accurate lower bound for the expected non-zero loadings (i.e., *Q*), making it impossible to tune *λ*_*L*_. Thus, we advocate the use of IS. It is possible that a hybrid method combining IS and stability selection may perform better than IS. For example, one first uses IS to decide the total number of non-zero loadings and then uses stability selection given the total number of non-zero loadings. Further examination on this idea is needed.

We focused on determining the status of the components (i.e., common/distinctive structure) and their level of sparseness. Another important issue that remains to be fully understood is the selection of the number of components *R*. Because the goal of this article is to understand variable selection methods for the component loading matrices, the selection of *R* is beyond the scope of this article. For interested readers, we refer to Bro, Kjeldahl, Smilde, and Kiers^47^, Gu and Van Deun^18^, and Måge, Smilde, and van der Kloet^48^. We believe that more studies are needed to evaluate the performance of model selection methods for determining *R* and the performance of variable selection. This may be done sequentially (i.e., first determining *R* and then, given *R*, performing variable selection) but also simultaneously (for example, using the index of sparseness to determine *R* and to perform variable selection at the same time). Finally, we call for studies on comparing the performance of variable selection methods in regularized models. The six variable selection methods studied in this article originated in sparse PCA literature. Therefore, we suspect that stability selection and IS would still outperform the other five methods in the sparse PCA settings. However, we are not aware of any study that compares variable selection methods in sparse PCA.

Admittedly, the six methods studied in this article do not constitute an exhaustive list of all possible variable selection methods for regularized SCA. Other variable selection methods exist, such as the method by Qi, Luo, and Zhao^49^, the information criterion by Chen and Chen^43^, and the numerical convex hull based method^50^, but they cannot be readily adapted to be used together with regularized SCA. These methods are promising though, and therefore require full attention in separate articles.

## Methods

### Regularized SCA

Let ${{\bf{X}}}_{k}\in { {\mathcal R} }^{I\times {J}_{k}},(k=1,2,\ldots ,K)$ denote the *k*th data block with *I* rows representing subjects, objects, or experimental conditions measured on *J*_*k*_ variables. One may notice that *I* does not have a subscript *k*, meaning that all *K* data blocks are to be analyzed jointly with respect to the same *I* subjects, objects, or experimental conditions. Each data block may have a different set of variables. Let ${{\bf{X}}}_{C}\in { {\mathcal R} }^{I\times {\sum }_{k}{J}_{k}}$ denote the concatenated data matrix, which is obtained by concatenating **X**_*k*_ s with respect to rows (i.e., ${{\bf{X}}}_{C}\equiv [{{\bf{X}}}_{1},\ldots ,{{\bf{X}}}_{K}]$). Note that *I* may be much smaller than *J*_*k*_ (i.e., high-dimensional data). Let ${\bf{T}}\in { {\mathcal R} }^{I\times R}$ denote the component score matrix, and let ${{\bf{t}}}_{r},(r=1,\ldots ,R)$ denote the *r*th column in **T**. Let ${{\bf{P}}}_{k}\in { {\mathcal R} }^{{J}_{k}\times R}$ denote the component loading matrix for the *k*th data block, and let ${{\bf{p}}}_{r}^{k},(k=1,\ldots ,K;r=1,\ldots ,R)$ denote the *r*th column in **P**_*k*_. Regularized SCA performs data integration by means of solving the following minimization problem,

10 $$\mathop{{\rm{\min }}}\limits_{{\bf{T}},{{\bf{P}}}_{k}}\,\sum _{k}\,\parallel {{\bf{X}}}_{k}-{\bf{T}}{{\bf{P}}}_{k}^{T}{\parallel }_{2}^{2}+{\lambda }_{L}\,\sum _{k}\,\parallel {{\bf{P}}}_{k}{\parallel }_{1}+{\lambda }_{G}\,\sum _{k}\,\sqrt{{J}_{k}}\parallel {{\bf{P}}}_{k}{\parallel }_{2},$$

subject to

$${{\bf{T}}}^{T}{\bf{T}}={\bf{I}};{\lambda }_{L},{\lambda }_{G}\ge 0.$$

Regularized SCA performs dimension reduction by imposing a pre-defined number of components, denoted by *R* ($R\le \,{\rm{\min }}(I,{\Sigma }_{k}{J}_{k})$; for details on deciding *R*, see Gu and Van Deun^18^). ${\sum }_{k}\,\parallel {{\bf{P}}}_{k}{\parallel }_{1}={\sum }_{k}\,{\sum }_{{j}_{k},r}\,|{p}_{{j}_{k}r}|$ is the Lasso penalty^16^, and its corresponding tuning parameter is *λ*_*L*_. ${\sum }_{k}\,\sqrt{{J}_{k}}\,\parallel {{\bf{P}}}_{k}{\parallel }_{2}={\sum }_{k}\,\sqrt{{J}_{k}\,{\sum }_{{j}_{k},r}\,({p}_{{j}_{k}r}^{2})}$ is the Group Lasso penalty^17^, and its corresponding tuning parameter is *λ*_*G*_. Note that if ${\lambda }_{L}=0$ and ${\lambda }_{G}=0$, Eq. 10 reduces to a least squares minimization problem. As a side note, before performing the regularized SCA analysis, all columns in **X**_*k*_ may be mean-centered and scaled to norm one or to ${J}_{k}^{-\mathrm{1/2}}$ in order to give all blocks - even those that contain relatively few variables - equal weight; This procedure is referred to as data pre-processing. However, one may notice that in Eq. 10 the Group Lasso penalty is also weighted by $\sqrt{{J}_{k}}$. Thus, it is likely that, when data are scaled to ${J}_{k}^{-\mathrm{1/2}}$, Eq. 10 would favor data blocks with fewer variables, because the Group Lasso penalty takes $\sqrt{{J}_{k}}$ into account. In addition, because in this study we are interested in identifying the associations between (some) variables across data blocks, penalties are imposed on the component loading matrix^19,46^. **T** is assumed to be the same for all *K* data blocks, and therefore it serves as a “bridge” linking all data blocks. Information shared among all data blocks or unique to some blocks, such as the loadings in Table 2, is obtained by estimating the component loading matrix ${{\bf{P}}}_{k},(k=1,2,\ldots ,K)$. Assuming **T** is known, we may further reduce Eq. 10 to

11 $$\mathop{{\rm{\min }}}\limits_{{{\bf{p}}}_{r}^{k}}\,{\Vert {{\bf{X}}}_{k}^{T}-\mathop{\sum }\limits_{r=1}^{R}\,{{\bf{p}}}_{r}^{k}{{\bf{t}}}_{r}^{T}\Vert }_{2}^{2}+{\lambda }_{L}\,\mathop{\sum }\limits_{r=1}^{R}\,\parallel {{\bf{p}}}_{r}^{k}{\parallel }_{1}+{\lambda }_{G}\sqrt{{J}_{k}}\,\mathop{\sum }\limits_{r=1}^{R}\,\parallel {{\bf{p}}}_{r}^{k}{\parallel }_{2}\mathrm{}.$$

Let $\hat{{\bf{T}}}$ denote the estimated component score matrix based on Eq. 10, and let ${\hat{{\bf{P}}}}_{k}$ denote the estimated component loading matrix for the *k*th data block. Further, Let ${\hat{{\bf{P}}}}_{C}\in { {\mathcal R} }^{({\sum }_{k}{J}_{k})\times R}$ denote the concatenated estimated component loading matrix, which is obtained by concatenating all ${\hat{{\bf{P}}}}_{k}\,{\rm{s}}$ with respect to the columns (i.e., ${\hat{{\bf{P}}}}_{C}\equiv {[{\hat{{\bf{P}}}}_{1}^{T},\ldots ,{\hat{{\bf{P}}}}_{K}^{T}]}^{T}$). The algorithm for estimating Eq. 10 requires an alternating procedure where $\hat{{\bf{T}}}$ and ${\hat{{\bf{P}}}}_{C}$ are estimated iteratively. Given ${\hat{{\bf{P}}}}_{C}$, $\hat{{\bf{T}}}$ is obtained by computing $\hat{{\bf{T}}}={\bf{V}}{{\bf{U}}}^{T}$, where **U**$\Sigma$**V**^*T*^ is the SVD of ${{\bf{P}}}_{C}^{T}{{\bf{X}}}_{C}^{T}$. Given $\hat{{\bf{T}}}$, ${\hat{{\bf{P}}}}_{C}$ is obtained by estimating ${{\bf{p}}}_{r}^{k}(k=1,2,\ldots K;r=1,2,\ldots ,R)$ in Eq. 11 ^18^:

12 $${\hat{{\bf{p}}}}_{r}^{k}={[\frac{1}{2}-\frac{{\lambda }_{G}\sqrt{{J}_{k}}}{2\parallel {\mathscr S}\mathrm{(2}{{\bf{X}}}_{k}^{T}{{\bf{t}}}_{r},{\lambda }_{L}){\parallel }_{2}}]}_{+}{\mathscr S}\mathrm{(2}{{\bf{X}}}_{k}^{T}{{\bf{t}}}_{r},{\lambda }_{L}\mathrm{)}.$$

In Eq. 12, ${\mathscr S}(\cdot )$ denotes the soft-thresholding operator. The operator [*x*]_+_ is defined as ${[x]}_{+}=x$, if $x > 0$, and ${[x]}_{+}=0$, if $x\le 0$. For details of the estimation procedure, see Algorithm 1 of Gu and Van Deun^18^.

Information regarding the position of non-zero/zero loadings in **P**_*C*_ may be known a priori. For example, Bolasso and stability selection procedures, which will be discussed shortly, can be used to identify the position of non-zero/zero loadings. Once the position of non-zero/zero loadings is identified, one uses regularized SCA with ${\lambda }_{L}={\lambda }_{G}=0$ to re-estimate the non-zero loadings in **P**_*C*_ while keeping the zero loadings fixed throughout the estimation procedure. For details of the estimation procedure, see Algorithm 2 of Gu and Van Deun^18^.

### Variable selection methods

The variable selection methods discussed in this article can be categorized into two groups. The first group, including CV with “one-standard-error” rule, rdCV, BIC criterion, and IS, aims at identifying the optimal *λ*_*L*_ and *λ*_*G*_ for Eq. 10. Once the optimal *λ*_*L*_ and *λ*_*G*_ are obtained, one re-estimates the model by using the optimal *λ*_*L*_ and *λ*_*G*_. The second group, including the Bolasso with CV and stability selection, aims at identifying the position of non-zero/zero loadings in **P**_*C*_ through repeated sampling. Once the position of non-zero/zero loadings is identified, one re-estimates the non-zero loadings while keeping the zero loadings fixed at zero. In the remainder of this article, we assume that the number of components *R* is known. To identify *R* in practice, one may use the Variance Accounted For (VAF) method^9,10^ and the PCA-GCA method^14^. Both methods are included in the R package “RegularizedSCA”^20^ (for details on how to use the two methods, see Gu and Van Deun^18^). We remind the reader that more research is needed for fully understanding how to identify *R*.

#### CV with “one-standard-error” rule

Given a set of *λ*_*L*_ s (consisting of evenly spaced increasing values ranging from a value close to zero, say, 0.000001, to the smallest value making ${\hat{{\bf{P}}}}_{C}={\bf{0}}$), denoted by ${\Lambda }_{L}$, and a set of *λ*_*G*_ s (also consisting of evenly spaced increasing values ranging from a value close to zero to the smallest value making ${\hat{{\bf{P}}}}_{C}={\bf{0}}$), denoted by ${\Lambda }_{G}$, the algorithm searches through a grid of *λ*_*L*_ s and *λ*_*s*_ s (i.e., the Cartesian product of ${\Lambda }_{L}$ and ${\Lambda }_{G}$). For each combination of *λ*_*L*_ and *λ*_*G*_, denoted by $({\lambda }_{L},{\lambda }_{G})$, the algorithm conducts *K*-fold CV. Take 10-fold CV for example, 10% of the data cells in **X**_*C*_ are replaced with missing values, and afterwards, missing values in each column are replaced with the mean of that column. The algorithm then computes the mean squared prediction errors (MSPE)^51^ for each $({\lambda }_{L},{\lambda }_{G})$. (Suppose a *Q*-fold CV ($Q=1,\ldots ,q,\ldots Q$) is performed. Let ${{\bf{X}}}_{k}^{(q)}$ denote the data from the *k*th block for the *q*th fold. Let ${\hat{{\bf{P}}}}_{k}^{(q)}$ denote the estimated component loading matrix for the *k*th data block for the *q*th fold. Let ${\hat{{\bf{T}}}}^{(q)}$ denote the estimated component score matrix for the *q*th fold. Then *MSPE* is ${\Sigma }_{q}\,{\Sigma }_{k}\,\parallel {{\bf{X}}}_{k}^{(q)}-{\hat{{\bf{T}}}}^{(q)}{({\hat{{\bf{P}}}}_{k}^{(q)})}^{T}{\parallel }_{2}^{2}/Q$). Let $MSP{E}_{({\lambda }_{L},{\lambda }_{G})}$ denote the MSPE given $({\lambda }_{L},{\lambda }_{G})$. Let $({\lambda }_{L}^{* },{\lambda }_{G}^{* })$ denote the pair that generates the smallest MSPE across all pairs of $({\lambda }_{L},{\lambda }_{G})\,{\rm{s}}$, and let $S{E}_{({\lambda }_{L}^{* },{\lambda }_{G}^{* })}$ denote the standard error of $MSP{E}_{({\lambda }_{L}^{* },{\lambda }_{G}^{* })}$. Applying the “one-standard-error” rule^26^, the algorithm searches for the optimal pair, denoted by $({\lambda }_{L}^{o},{\lambda }_{G}^{o})$, such that its MSPE, $MSP{E}_{({\lambda }_{L}^{o},{\lambda }_{G}^{o})}$, is closest to but not larger than $MSP{E}_{({\lambda }_{L}^{* },{\lambda }_{G}^{* })}+S{E}_{({\lambda }_{L}^{* },{\lambda }_{G}^{* })}$. As a side note, in the simulation, the algorithm searched the optimal pair whose MSPE was closest to (i.e., could be slightly larger or smaller than) $MSP{E}_{({\lambda }_{L}^{\ast },{\lambda }_{G}^{\ast })}+S{E}_{({\lambda }_{L}^{\ast },{\lambda }_{G}^{\ast })}$. In the simulation, we used 5-fold CV.

#### Repeated double cross-validation (rdCV)

**Figure 11:**
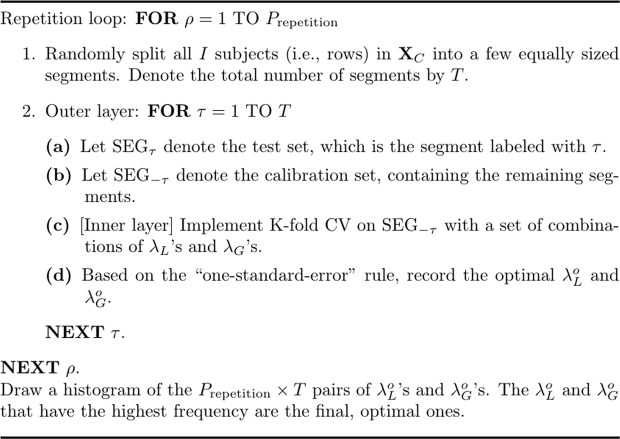
The algorithm of the rdCV.

The rdCV^27^, as its name would suggest, is an algorithm that performs double CV repeatedly. Double CV consists of two so-called “layers”, and at each layer a CV is executed. Figure 11 presents a sketch of the algorithm. In the $\rho$th repetition ($\rho =1,\ldots ,{P}_{{\rm{repetition}}}$), the concatenated dataset, **X**_*C*_, is randomly split into *T* segments with a (nearly) equal sample size; that is, each segment contains (roughly) the same number of subjects/objects/experimental conditions. The $\tau$th segment, denoted by ${{\rm{SEG}}}_{\tau }$ ($\tau =1,\ldots ,T$), is used as the test set, and the remaining segments constitute the calibration set, denoted by ${{\rm{SEG}}}_{-\tau }$. The algorithm then executes CV with “one-standard-error” rule on ${{\rm{SEG}}}_{-\tau }$ and generates the optimal $({\lambda }_{L}^{o},{\lambda }_{G}^{o})$ for ${{\rm{SEG}}}_{-\tau }$. Thus, in total, ${P}_{{\rm{repetition}}}\times T$ pairs of $({\lambda }_{L}^{o},{\lambda }_{G}^{o})\,{\rm{s}}$ are generated. Note that, in Fig. 11, one may add an extra step after Step (d): In this extra step, one may calculate the MSPE, which provides information for selecting optimal tuning parameters. But Filzmoser, Liebmann, and Varmuza^27^ suggested that the extra step might be omitted: One may simply use a histogram or a frequency table for the ${P}_{{\rm{repetition}}}\times T$ pairs of ${\lambda }_{L}^{o}\,{\rm{s}}$ and ${\lambda }_{G}^{o}\,{\rm{s}}$ and choose the ${\lambda }_{L}^{o}$ and ${\lambda }_{G}^{o}$ that have been generated most frequently by the algorithm. In the simulation, we let the algorithm choose the most frequently generated ${\lambda }_{L}^{o}$ and ${\lambda }_{G}^{o}$ separately, which was more efficient computationally. In addition, we used 5-fold CV for the inner layer, and for the outer layer, we set the number of segment $T=2$ and the number of repetition ${P}_{{\rm{repetition}}}=50$.

#### The BIC criterion

Given a set of *λ*_*L*_ s (consisting of evenly spaced increasing values ranging from a value close to zero, say, 0.000001, to the smallest value making ${\hat{{\bf{P}}}}_{C}={\bf{0}}$), denoted by ${\Lambda }_{L}$, and a set of *λ*_*G*_ s (also consisting of evenly spaced increasing values ranging from a value close to zero to the smallest value making ${\hat{{\bf{P}}}}_{C}={\bf{0}}$), denoted by ${\Lambda }_{G}$, the algorithm searches through a grid of *λ*_*L*_ s and *λ*_*G*_ s (i.e., the Cartesian product of ${\Lambda }_{L}$ and ${\Lambda }_{G}$). For each combination of *λ*_*L*_ and *λ*_*G*_, denoted by $({\lambda }_{L},{\lambda }_{G})$, the algorithm computes the BIC.

The BIC criterion used in this article is based on two BIC criteria in the sparse PCA literature, one proposed by Croux, Filzmoser, and Fritz^34^ and the other one by Guo, James, Levina, Michailidis, and Zhu^35^. We define the variance of the residual matrix if there would be no sparseness in $\hat{{\bf{P}}}$, denoted by *V*, as $V=\parallel {{\bf{X}}}_{C}-{\hat{{\bf{T}}}}^{(sca)}{({\hat{{\bf{P}}}}_{C}^{(sca)})}^{T}{\parallel }_{2}^{2}$, where ${\hat{{\bf{T}}}}^{(sca)}$ and ${\hat{{\bf{P}}}}_{C}^{(sca)}$ are obtained from the traditional simultaneous component model without Lasso and Group Lasso penalties. We define the variance of the residual matrix given *λ*_*L*_ and *λ*_*G*_, denoted by $\tilde{V}$, as $\tilde{V}=\parallel {{\bf{X}}}_{C}-\hat{{\bf{T}}}{\hat{{\bf{P}}}}_{C}^{T}{\parallel }_{2}^{2}$, where $\hat{{\bf{T}}}$ and ${\hat{{\bf{P}}}}_{C}$ are obtained from Eq. 10. We define the degrees of freedom given *λ*_*L*_ and *λ*_*G*_, denoted by $df({\lambda }_{L},{\lambda }_{G})$, as the number of non-zero loadings in ${\hat{{\bf{P}}}}_{C}$. Then the BIC criterion adjusted for regularized SCA, given *λ*_*L*_ and *λ*_*G*_, based on Croux *et al*. is

13 $$BIC({\lambda }_{L},{\lambda }_{G})=\frac{\tilde{V}}{V}+df({\lambda }_{L},{\lambda }_{G})\frac{log(I)}{I},$$

and the BIC criterion adjusted for the regularized SCA method based on Guo *et al*. is

14 $$BIC({\lambda }_{L},{\lambda }_{G})=\frac{I\tilde{V}}{V}+df({\lambda }_{L},{\lambda }_{G})log(I\mathrm{)}.$$

Notice that the BIC in Eq. 14 is exactly *I* times the BIC in Eq. 13. Thus, the two methods are in fact equivalent. Then, the optimal tuning parameter values, $({\lambda }_{L}^{o},{\lambda }_{G}^{o})$, are the ones that generate the lowest BIC.

#### Index of Sparseness (IS)

Given a set of *λ*_*L*_ s (consisting of evenly spaced increasing values ranging from a value close to zero, say, 0.000001, to the smallest value making ${\hat{{\bf{P}}}}_{C}={\bf{0}}$), denoted by ${\Lambda }_{L}$, and a set of *λ*_*G*_ s (also consisting of evenly spaced increasing values ranging from a value close to zero to the smallest value making ${\hat{{\bf{P}}}}_{C}={\bf{0}}$), denoted by ${\Lambda }_{G}$, the algorithm searches through a grid of *λ*_*L*_ s and *λ*_*s*_ s (i.e., the Cartesian product of ${\Lambda }_{L}$ and ${\Lambda }_{G}$). For each combination of *λ*_*L*_ and *λ*_*G*_, denoted by $({\lambda }_{L},{\lambda }_{G})$, the algorithm computes the IS.

We define the total variance in **X**_*C*_, denoted by *V*_*o*_, as ${V}_{o}=\parallel {{\bf{X}}}_{C}{\parallel }_{2}^{2}$. The unadjusted variance assuming no penalty (i.e., ${\lambda }_{L}={\lambda }_{G}=0$), denoted by *V*_*s*_, is defined as ${V}_{s}=\parallel {\hat{{\bf{T}}}}^{(sca)}{({\hat{{\bf{P}}}}_{C}^{(sca)})}^{T}{\parallel }_{2}^{2}$. Finally, the adjusted variance, denoted by *V*_*a*_, is defined as ${V}_{a}=\parallel \hat{{\bf{T}}}{\hat{{\bf{P}}}}_{C}^{T}{\parallel }_{2}^{2}$, where $\hat{{\bf{T}}}$ and ${\hat{{\bf{P}}}}_{C}$ are obtained from Eq. 10 (i.e., ${\lambda }_{L}\ne 0$ and ${\lambda }_{G}\ne 0$). Let #_*o*_ denote the total number of zero loadings in ${\hat{{\bf{P}}}}_{C}$. Then IS, according to Gajjar, Kulahci, and Palazoglu^28^ and Trendafilov^29^, is

15 $$IS=\frac{{V}_{a}{V}_{s}}{{V}_{o}^{2}}\times \frac{{\#}_{o}}{({\sum }_{k}\,{J}_{k})\times R}.$$

The optimal tuning parameter values, $({\lambda }_{L}^{o},{\lambda }_{G}^{o})$, are the ones that generate the largest IS.

#### Bolasso with CV

**Figure 12:**
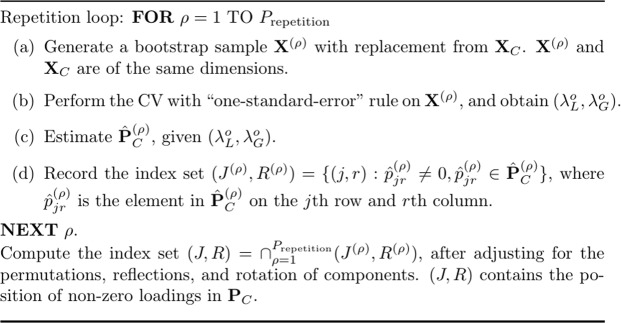
The algorithm of the Bolasso with CV.

Bolasso, originally proposed by Bach^31^, has been extended to a hybrid procedure combining the original Bolasso with CV^32,33^ for stably selecting variables in Lasso regression. Figure 12 presents the algorithm of the Bolasso with CV. In essence, the Bolasso is a bootstrapping procedure. For each bootstrap sample, regularized SCA with K-fold CV is executed, generating the optimal tuning parameters, $({\lambda }_{L}^{o},{\lambda }_{G}^{o})$ based on the “one-standard-error” rule. Afterwards, ${\hat{{\bf{P}}}}_{C}$ is obtained given $({\lambda }_{L}^{o},{\lambda }_{G}^{o})$. Let *P*_repetition_ denote the total number of repetitions. Then in total *P*_repetition_ ${\hat{{\bf{P}}}}_{C}\,{\rm{s}}$ are generated. The algorithm then compares the *P*_repetition_ ${\hat{{\bf{P}}}}_{C}\,{\rm{s}}$, checks which loadings have been estimated to be not zeros for *P*_repetition_ times, and records the corresponding index set. As a result, an index set containing the position of non-zero loadings is obtained. Finally, ${\hat{{\bf{P}}}}_{C}$ and $\hat{{\bf{T}}}$ are estimated given the index set. One may notice that because of the invariance of the regularized SCA solution under permutations of components^18^, the ${\hat{{\bf{P}}}}_{C}\,{\rm{s}}$ must first be adjusted according to a reference matrix by using the Tucker congruence^42^ (for details, see the R script provided in the supplementary material). As a side note, in the simulation, we used 5-fold CV and let ${P}_{{\rm{repetition}}}=50$.

#### Stability selection

**Figure 13:**
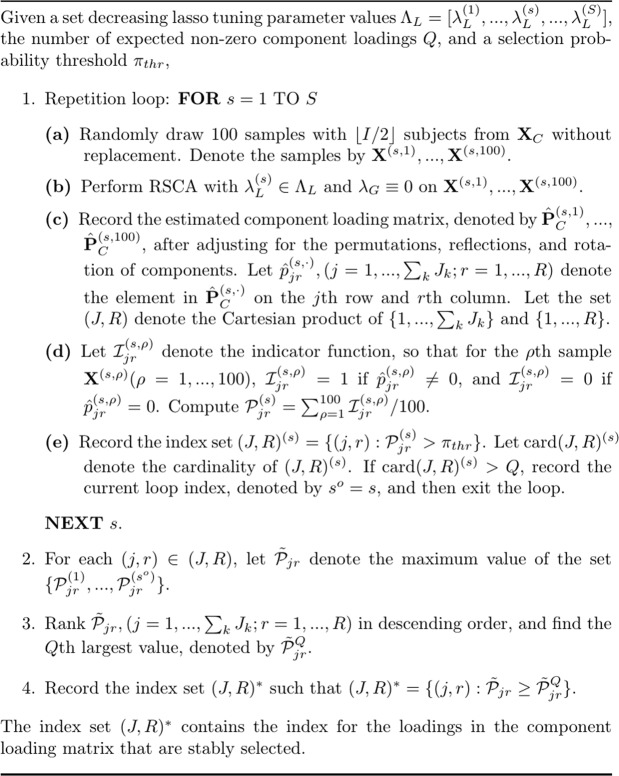
The algorithm of stability selection adjusted for regularized SCA.

Stability selection^25^ was demonstrated for variable selection in regression analysis and graphical models based on the Lasso. To use this method for regularized SCA, we have made a few adjustments and present the algorithm in Fig. 13. The algorithm goes through a set of *S* Lasso tuning parameter values with decreasing order, denoted by ${\Lambda }_{L}=[{\lambda }_{L}^{(1)},{\lambda }_{L}^{(2)},\ldots ,{\lambda }_{L}^{(s)},\ldots ,{\lambda }_{L}^{(S)}]$, $({\lambda }_{L}^{(1)} > {\lambda }_{L}^{(2)} > \cdots  > {\lambda }_{L}^{(s)} > \cdots  > {\lambda }_{L}^{(S)})$, indexed by $s=1,2,\ldots ,S$. ${\lambda }_{L}^{\mathrm{(1)}}$ is fixed at the minimum value that makes ${\hat{{\bf{P}}}}_{C}\equiv {\bf{0}}$. Given the *s*th value, ${\lambda }_{L}^{(s)}$, the algorithm works as follows. First, 100 samples with $\lfloor I\mathrm{/2}\rfloor$ subjects (i.e., rows) from **X**_*C*_ are randomly drawn without replacement. For each sample created, regularized SCA with ${\lambda }_{L}^{(s)}$ and ${\lambda }_{G}=0$ is applied. Therefore, the algorithm generates 100 ${\hat{{\bf{P}}}}_{C}\,{\rm{s}}$. Because of the invariance of regularized SCA solution under permutations of components, the ${\hat{{\bf{P}}}}_{C}\,{\rm{s}}$ are adjusted according to a common reference matrix by using the Tucker congruence (for details, see the R script in the supplementary material). Then, the algorithm counts the number of times that the same loading is estimated to be a non-zero loading across the 100 ${\hat{{\bf{P}}}}_{C}\,{\rm{s}}$, which is then divided by 100, resulting in the selection probability for that loading (see Step 1(d) in Fig. 13). As a result, each component loading has a selection probability, which is then compared to a pre-defined selection probability threshold *π*_*thr*_, and the loadings for which the selection probabilities lower than *π*_*thr*_ are constrained to be zero loadings. The error control theorem proposed by Meinshausen and Bühlmann^25^ (Theorem 1, p. 7) adjusted for the regularized SCA model is

16 $$EV\le \frac{1}{2{\pi }_{thr}-1}\times \frac{{Q}^{2}}{R\,{\sum }_{k}\,{J}_{k}},$$

where *EV* denotes the expected number of falsely selected variables, *Q* denotes the expected non-zero loadings, and $R\,{\sum }_{k}\,{J}_{k}$ is the total number of loadings. We notice that, when Gu and Van Deun^19^ applied stability selection in their study on regularized SCA, they failed to recognize the problem of Eq. 16: When used for regularized SCA, the lower bound for *Q* produced by Eq. 16 is not strict enough, making it difficult to tune ${\Lambda }_{L}$. To explain, we use the first simulation study in the Results section as an example and consider the situation of ${{\bf{X}}}_{1}=\{{x}_{ij}\}\in { {\mathcal R} }^{20\times 120}\,{\rm{and}}\,{{\bf{X}}}_{2}=\{{x}_{ij}\}\in { {\mathcal R} }^{20\times 30}$ and 50% of loadings in ${{\bf{p}}}_{1}^{1}$, ${{\bf{p}}}_{1}^{2}$, ${{\bf{p}}}_{2}^{2}$, and ${{\bf{p}}}_{3}^{1}$ are zero loadings. In this case, the total number of non-zero loadings is 150, and the total number of loadings is $R\,{\sum }_{k}\,{J}_{k}=3\times 150=450$. If we use Eq. 16 and let $EV=1$, and ${\pi }_{thr}=0.9$, then $Q\ge 19$, which is much smaller than 150 (i.e, the total number of non-zero loadings). Thus, using Eq. 16 to tune ${\Lambda }_{L}$ is likely to generate a component loading matrix that is too sparse. In this article, the algorithm tunes ${\Lambda }_{L}$ by using the number of expected non-zero component loadings *Q*, which is assumed known a priori (see Step 1(e) in Fig. 13). Thus, given ${\lambda }_{L}^{(s)}$, if the total number of loadings with selection probability not lower than *π*_*thr*_ is equal to or larger than *Q*, then the algorithm ignores the remaining Lasso tuning parameter values $[{\lambda }_{L}^{(s+1)},\ldots ,{\lambda }_{L}^{(S)}]$. Assume the algorithm stops at ${\lambda }_{L}^{(s)}$, then for each loading, there are *s* selection probabilities generated based on $[{\lambda }_{L}^{(1)},\ldots ,{\lambda }_{L}^{(s)}]$. The algorithm records the maximum selection probability across the *s* selection probabilities for each loading, ranks the loadings in descending order according to their associated maximum selection probabilities, and picks the loadings whose maximum probabilities belong to the first *Q* maximum probabilities (see steps 2, 3, and 4 in Fig. 13). Finally, the selected loadings are re-estimated, while the remaining loadings are fixed at zero. As a side note, in the simulation, we set ${\pi }_{thr}=0.6$. Also in the simulation, *Q* was known, which was the total number of non-zero loadings in ${{\bf{P}}}_{C}^{true}$, but this is unrealistic in practice.

## Supplementary information

R script
