# Supplementary material for: Variable Selection in the Regularized Simultaneous Component Analysis Method for Multi-Source Data Integration
Source: Sci Rep. 2019 Dec 9;9:18608. doi: 10.1038/s41598-019-54673-2 (PMC6901488; doi:10.1038/s41598-019-54673-2)
Supplement: Supplementary file 1 — R script [file 41598_2019_54673_MOESM1_ESM.pdf]

Supplementary material - R script for replication of the studies

1. function: repeated double CV

```
####### parameters
########################################################################

# DATA: concatenated data matrix

# Jk: number of variables (a vector, the elements of which correspond to the datablocks)

# R: number of components

# LassoSequence: A sequence of Lasso tuning parameters

# GLassoSequence: A sequence of Group Lasso tuning parameters

# n_rep: number of repetitions

# n_seg: number of segments

# N_cores: number of cores (for parallel computing)

##########################################################################################
##


M1_repeatedDoubleCV <- function(DATA, R, Jk, N_cores, LassoSequence,  GLassoSequence, n_rep,
n_seg, NRSTARTS, nfolds, MaxIter){

  #library(RegularizedSCA)

  #library(foreach)

  #library(snow)

  #library(doSNOW)

  #library(doRNG)


  if(missing(N_cores)){

    N_cores = 1

  }


  if(missing(n_rep)){
```

```
  n_rep <- 10
}

if(missing(n_seg)){
  n_seg <- 3
}


if(missing(LassoSequence)){
  LassoSequence = seq(0.001, RegularizedSCA::maxLGlasso(DATA, Jk, R)$Lasso, length.out = 10)
}

if(missing(GLassoSequence)){
  GLassoSequence = seq(0.001, RegularizedSCA::maxLGlasso(DATA, Jk, R)$Glasso, length.out = 10)
}

if(missing(NRSTARTS)){
  NRSTARTS = 1
}


DATA <- data.matrix(DATA)
nsub <- dim(DATA)[1]


################################ Repeated Double CV
###########################################
```

```
perc_test <- 1/n_seg

cl <- snow::makeCluster(N_cores)
doSNOW::registerDoSNOW(cl)

#note that set.seed() and %dorng% ensure that parallel computing generates reproducable results.
sim_result <- foreach::foreach(r = 1:n_rep, .combine='cbind') %dorng% {

  Flag_person <- 0  # this, together with the following while(min_person=0), is to ensure that
  # the number of subject in testset > number of components. !!! important
  randindex <- stats::runif(nsub, 0, 1)
  testset_indexsize <- array(NA, n_seg) #note that each element in the array will be compared to R.
  while(Flag_person == 0){
   for(i in 1:n_seg){
    #randindex <- runif(nsub, 0, 1)
    testset_index <- ((i - 1) * perc_test < randindex) & (randindex < i * perc_test)
    testset_indexsize[i] <- sum(testset_index)
   }
   if(sum(testset_indexsize<=R)>0){
    randindex <- stats::runif(nsub, 0, 1)  #The number of persons should be > than R. if not, resample.
   }else{
    Flag_person <- 1
   }
  }

  #e_hat <- array()
  OptimumLasso <- array()
  OptimumGLasso <- array()
  for(i in 1:n_seg){
```

```
      testset_index <- ((i - 1) * perc_test < randindex) & (randindex < i * perc_test)

      #testset <- DATA[testset_index, ]

      calibset <- DATA[!testset_index, ]


      results_innerloop <- RegularizedSCA::cv_sparseSCA(calibset, Jk, R, MaxIter, NRSTARTS,
LassoSequence, GLassoSequence, nfolds, method = "component")

      OptimumLasso[i] <- results_innerloop$RecommendedLambda[1]

      OptimumGLasso[i] <- results_innerloop$RecommendedLambda[2]

      #estimatedP <- results_innerloop$P_hat

      #A <- t(estimatedP) %*% t(testset)

      #SVD_DATA <- svd(A, R, R)

      #estimatedT <- SVD_DATA$v %*% t(SVD_DATA$u)

      #e_hat[i] <- sum((testset - estimatedT %*% t(estimatedP))^2)  COMMENTS: According to Filzmoser,
Liebmann, & Varmuza (2009), no need to

      #compute MSE. One may simply check the frequency table of the optimal lasso and glasso tuning
parameter values. This is why I omit the MSE steps.


    }


    final_sim <- cbind(OptimumLasso, OptimumGLasso)


    #final_sim[[3]] <- e_hat

    return(final_sim)

}


snow::stopCluster(cl)


#results <- list()

#results$Lasso <- OptimumLasso
```

```
#results$GLasso <- OptimumGLasso

#results$e_hat <- E_hat


return_tables <- list(Lasso = table(sim_result[, colnames(sim_result) == "OptimumLasso"]),

                      GroupLasso = table(sim_result[, colnames(sim_result) == "OptimumGLasso"]))

return(return_tables)

}
```

2. function: BIC and IS

```
####### parameters
########################################################################

# DATA: concatenated data matrix

# Jk: number of variables (a vector, the elements of which correspond to the datablocks)

# R: number of components

# LassoSequence: A sequence of Lasso tuning parameters

# GLassoSequence: A sequence of Group Lasso tuning parameters

#####################################################################################
######


M2_BIC_IS <- function(DATA, Jk, R, LassoSequence, GLassoSequence, NRSTARTS, MaxIter){


  DATA <- data.matrix(DATA)

  n_sub <- dim(DATA)[1]


  if(missing(LassoSequence)){

    LassoSequence = seq(0.001, RegularizedSCA::maxLGlasso(DATA, Jk, R)$Lasso, length.out = 20)

  }
```

```
  if(missing(GLassoSequence)){
    GLassoSequence = seq(0.001, RegularizedSCA::maxLGlasso(DATA, Jk, R)$Glasso, length.out = 20)
  }

  if(missing(NRSTARTS)){
    NRSTARTS = 5
  }

  VarSelect0 <- RegularizedSCA::sparseSCA(DATA, Jk, R, LASSO = 0, GROUPLASSO = 0, MaxIter,
NRSTARTS, method = "component")
  P_hat0 <- VarSelect0$Pmatrix
  T_hat0 <- VarSelect0$Tmatrix

  V_0 <- sum((DATA - T_hat0%*%t(P_hat0))^2)  # this is for BIC_Croux and BIC_GUO
  error_var <- V_0 / n_sub  #this is for BIC_Guo

  V_oo <- sum(DATA^2)  # this is for Index of sparseness (IS)
  V_s <- sum((T_hat0%*%t(P_hat0))^2)  # this is for IS

  BIC_Croux <- matrix(NA, length(LassoSequence), length(GLassoSequence))
  BIC_Guo <- matrix(NA, length(LassoSequence), length(GLassoSequence))
  IS <- matrix(NA, length(LassoSequence), length(GLassoSequence))
  for(i in 1:length(LassoSequence)){
    for(j in 1:length(GLassoSequence)){

      VarSelect <- RegularizedSCA::sparseSCA(DATA, Jk, R, LASSO = LassoSequence[i], GROUPLASSO =
GLassoSequence[j], MaxIter, NRSTARTS = NRSTARTS, method = "component")
      P_hat <- VarSelect$Pmatrix
      T_hat <- VarSelect$Tmatrix
```

```
    V_tilde <- sum((DATA - T_hat %*% t(P_hat))^2)


    BIC_Croux[i, j] <- V_tilde / V_0 + sum(P_hat != 0) * log(n_sub) / n_sub  # this is the BIC index prop0sed by Croux et al.
    BIC_Guo[i, j] <- V_tilde / error_var + sum(P_hat != 0) * log(n_sub)      # this is the BIC index proposed by Guo et al.


    V_a <- sum((T_hat %*% t(P_hat))^2)  # this is for IS
    IS[i, j] <- V_a * V_s / V_oo^2 * sum(P_hat == 0) /(sum(Jk) * R)         # this is index of sparseness
  }
 }


 BIC_list <- list(Croux = BIC_Croux, GUO = BIC_Guo, IS = IS)
 return(BIC_list)
}
```

3. Bolasso

```
#Bolasso with CV


# Input parameters:
# DATA: Concatenated data matrix (!!! Not standardized! the matrix will be standardized after each resampling)
# Jk: A vector. Each element of this vector is the number of columns of a data block
# N_boots: number of bootstrap relicates
# R: The number of components (R>=2)
# LassoSequence: A vector of lasso tuning parameter values in accending order
# GlassoSequence: A vector of Group Lasso tuning parameter values in accending order
# N_cores: Number of cores for parallel computing
```

```
Bolasso_CV <- function(DATA, Jk, R, N_boots, LassoSequence, GLassoSequence, N_cores, NRSTARTS,
nfolds, MaxIter){

  #library(foreach)
  #library(doSNOW)
  #library(doRNG)
  #library(RegularizedSCA)

  DATA <- data.matrix(DATA)  #DATA should be pre-processed at this stage

  if(missing(LassoSequence)){
    LassoSequence = seq(0.001, RegularizedSCA::maxLGlasso(DATA, Jk, R)$Lasso, length.out = 20)
  }

  if(missing(GLassoSequence)){
    GLassoSequence = seq(0.001, RegularizedSCA::maxLGlasso(DATA, Jk, R)$Glasso, length.out = 20)
  }

  if(missing(NRSTARTS)){
    NRSTARTS = 1
  }

  n_persons <- nrow(DATA)
  person_index <- sample(1:n_persons, n_persons, replace = TRUE)
  Data_sample <- DATA[person_index, ]
  result <- RegularizedSCA::cv_sparseSCA(Data_sample, Jk, R, MaxIter, NRSTARTS, LassoSequence,
GLassoSequence, nfolds, method = "component")
  T_target <- result$T_hat              #We fix the estimated T matrix from the first resampled data.
                                        #All the estimated T matrix are to be compared to this estimated T.
```

```
                                #(This is due to permutation freedom)
  P_indexset <- result$P_hat
  P_indexset[which(P_indexset!=0)] <- 1  #non-zero loadings are marked as 1

  #resampling
  cl <- snow::makeCluster(N_cores)
  doSNOW::registerDoSNOW(cl)
  #note that set.seed() and %dorng% ensure that parallel computing generates reproducable results.
  sim_result <- foreach::foreach(i = 1:(N_boots-1), .combine='+') %dorng% {

    person_index <- sample(1:n_persons, n_persons, replace = TRUE)
    Data_sample <- DATA[person_index, ]
    result <- RegularizedSCA::cv_sparseSCA(Data_sample, Jk, R, MaxIter, NRSTARTS, LassoSequence,
GLassoSequence, nfolds, method = "component")
    T_result <- result$T_hat
    perm <- RegularizedSCA::TuckerCoef(T_target, T_result)$perm
    P_result <- result$P_hat[, perm]
    P_result[which(P_result!=0)] <- 1

    return(P_result)

  }
  snow::stopCluster(cl)

  P_indexset <- data.frame(sim_result) + P_indexset

  P_indexset[P_indexset != N_boots] <- 0  #Variables that have not been selected N_boots times are set
to be zero
```

```
  # Reestimate P and T, with 5 multi-starts
  Pout3d <- list()
  Tout3d <- list()
  LOSS <- array()
  LOSSvec <- list()

  for (n in 1:5) {
    VarSelectResult <- StrucSCA_withIndex(DATA, Jk, R, P_indexset = P_indexset, MaxIter)
    Pout3d[[n]] <- VarSelectResult$Pmatrix
    Tout3d[[n]] <- VarSelectResult$Tmatrix
    LOSS[n] <- VarSelectResult$Loss
    LOSSvec[[n]] <- VarSelectResult$Lossvec
  }
  k <- which(LOSS == min(LOSS))
  if (length(k) > 1) {
    pos <- sample(1:length(k), 1)
    k <- k[pos]
  }

  PoutBest <- Pout3d[[k]]  #this is the final, estimated P
  ToutBest <- Tout3d[[k]]  #this is the final, estimated T

  return_list <- list(P_hat = PoutBest, T_hat = ToutBest)
  return(return_list)

}
```

4. stability selection adjusted for RSCA

```
####### parameters
##############################################################################

# DATA: concatenated data matrix

# Jk: number of variables (a vector, the elements of which correspond to the datablocks)

# R: number of components

# LassoSequence: A sequence of Lasso tuning parameters

# N_loading: The total number of non-zero loadings in the P matrix

# Thr: threshold for the probability of each loading; the default is .9

# NRSTARTS: Number of random starts

# N_cores: number of CPU logical cores can be used

##########################################################################################
######


M4_StabSelection <- function(DATA, Jk, R, LassoSequence, N_loading, Thr, NRSTARTS, N_cores, nfolds,
MaxIter){


  DATA <- data.matrix(DATA)

  n_persons <- nrow(DATA)


  if(missing(LassoSequence)){

    LassoSequence = exp(seq(from = log(0.00000001), to = log(RegularizedSCA::maxLGlasso(DATA, Jk,
R)$Lasso), length.out = 100))

  }


    if(missing(NRSTARTS)){

    NRSTARTS = 5

  }


  if(missing(Thr)){

    Thr = .5
```

```
  }

  # need a target matrix for T, so that all the estimated T can be compared to it.
  result <- RegularizedSCA::cv_sparseSCA(DATA, Jk, R, MaxIter, NRSTARTS, LassoSequence,
GLassoSequence=0, nfolds, method = "component")
  T_target <- result$T_hat                #We fix the estimated T matrix. All the estimated P in the following
resampling procedure will be rotated
                                          #after comparing the estimated T with with T_target.
                                          #Arguably, using CV to generate T_target might not be a good idea. By the time
I'm writing this code, I have already
                                          #found out that index of sparseness works better. But CV is the most well known
method, so I use CV here.

  LassoSequence <- sort(LassoSequence, decreasing = T) # the largest value enters first

  P_prob <- list()
  #### this is for P_prob[[1]], which is when used to compare to P_prob[[j]] (j= 2, ...) so as to record the
highest probability across all j's (j=1,...)
  cl <- snow::makeCluster(N_cores)
  doSNOW::registerDoSNOW(cl)
  #note that set.seed() and %dorng% ensure that parallel computing generates reproducable results.
  sim_result <- foreach::foreach(i = 1:100, .combine='+') %dorng% {

    person_index <- sample(1:n_persons, n_persons/2, replace = F)
    result <- RegularizedSCA::sparseSCA(DATA[person_index, ], Jk, R, LASSO = LassoSequence[1],
GROUPLASSO = 0, MaxIter, NRSTARTS, method = "component")
    perm <- RegularizedSCA::TuckerCoef(T_target[person_index, ], result$Tmatrix)$perm
    P_result <- result$Pmatrix[, perm]
    P_result[which(P_result!=0)] <- 1

    return(P_result)
```

```
  }
  snow::stopCluster(cl)
  P_prob[[1]] <- data.frame(sim_result)/100
  P_final <- P_prob[[1]]  #P_final will be updated after each comparison (see below)
  j <- 2
  while(j <= length(LassoSequence)){  #note that it is not necessary to run j all the way to the length(LassoSequence), to some point, this algorthm will stop because enough non-zero loadings are gathered.
    cl <- snow::makeCluster(N_cores)
    doSNOW::registerDoSNOW(cl)
    #note that set.seed() and %dorng% ensure that parallel computing generates reproducable results.
    sim_result <- foreach::foreach(i = 1:100, .combine='+') %dorng% {

      person_index <- sample(1:n_persons, n_persons/2, replace = F)
      result <- RegularizedSCA::sparseSCA(DATA[person_index, ], Jk, R, LASSO = LassoSequence[j], GROUPLASSO = 0, MaxIter, NRSTARTS, method = "component")
      perm <- RegularizedSCA::TuckerCoef(T_target[person_index, ], result$Tmatrix)$perm
      P_result <- result$Pmatrix[, perm]
      P_result[which(P_result!=0)] <- 1

      return(P_result)

    }
    snow::stopCluster(cl)

    P_prob[[j]] <- data.frame(sim_result)/100

    index <- which((P_final < P_prob[[j]]), arr.ind = TRUE)  # which((P_final < P_prob[[j]]) == TRUE, arr.ind = TRUE) is also fine
```

```
    P_final[index] <- P_prob[[j]][index]  #update the P_final matrix, keep the highest probability

    if(sum(P_prob[[j]] >= Thr) > N_loading){
      j <- length(LassoSequence)  # just a way to skip the remaining Lasso values, since the total number of non-zero loadings already > N_loading prespecified by the user
    }

    j <- j+1
    print(j)
  }

  # Note that in the above code if(sum(P_prob[[j]] >= Thr) > N_loading), it could happen that at j, sum(P_prob[[j]]) >> N_loading. We would want to reduce the size of sum(P_prob[[j]]) close to N_loading
  thr_new <- sort(as.matrix(P_final), decreasing = TRUE)[N_loading] # this is the lowest probability whose corresponding loading should not be zero, ACCORDING TO N_loading
  if(dim(which(P_final== thr_new, arr.ind = TRUE))[1] > 1){
    print("It's likely that the total # of non-0 loadings in the final P_hat greatly exceeds N_loading!")  #this is problematic, in this case, more than one loading corresponds to the lowest probability.
  }

  P_final[which(P_final < thr_new, arr.ind = TRUE)] <- 0

  # Reestimate P and T, with 5 starts
  Pout3d <- list()
  Tout3d <- list()
  LOSS <- array()
  LOSSvec <- list()

  for (n in 1:NRSTARTS) {
    VarSelectResult <- StrucSCA_withIndex(DATA, Jk, R, P_indexset = P_final, MaxIter)
```

```
    Pout3d[[n]] <- VarSelectResult$Pmatrix
    Tout3d[[n]] <- VarSelectResult$Tmatrix
    LOSS[n] <- VarSelectResult$Loss
    LOSSvec[[n]] <- VarSelectResult$Lossvec
  }
  k <- which(LOSS == min(LOSS))
  if (length(k) > 1) {
    pos <- sample(1:length(k), 1)
    k <- k[pos]
  }

  PoutBest <- Pout3d[[k]]  #this is the final, estimated P
  ToutBest <- Tout3d[[k]]  #this is the final, estimated T

  return_list <- list(P_hat = PoutBest, T_hat = ToutBest)
  return(return_list)


}
```

5. generating data: 2 blocks

```
set.seed(1)

Perc0 = .5   #.3 or .5
PropNoise = 0.3 # .005, .3

I <- 20     # 20 or 100
J1 <- 120    # 40 or 120
J2 <- 30    # 10 or 30
```

```
Jk <- c(J1, J2)

R <- 3

N_dataset <- 20  #how many datasets to generate




###########################################################################################
#############################################

# A function for generating data

###########################################################################################
#############################################

Data_generation <- function(I, J1, J2, R, PropNoise, Perc0, N_dataset){


  Jk <- c(J1, J2)

  sumJk <- sum(J1 + J2)


  DATA1 <- matrix(rnorm(I*J1, mean = 0, sd = 1), I, J1)

  DATA2 <- matrix(rnorm(I*J2, mean = 0, sd = 1), I, J2)

  DATA <- cbind(DATA1, DATA2)


  svddata <- svd(DATA, R, R)

  Ttrue <- svddata$u

  PTrueC <- as.matrix(svddata$v) %*% diag(svddata$d[1:R])   #note that only the first R eigen values are
needed.


  PTrueCBlock1 <- PTrueC[1:J1,]

  PTrueCBlock2 <- PTrueC[(J1+1):(J1+J2),]


  PTrueCBlock1[, 2] <- 0
```

```
  PTrueCBlock2[, 3] <- 0

  v <- sample(1:J1, size = round(Perc0*J1), replace = F)
  PTrueCBlock1[, 3][v] <- 0
  v <- sample(1:J2, size = round(Perc0*J2), replace = F)
  PTrueCBlock2[, 2][v] <- 0
  PTrueC_bind <- rbind(PTrueCBlock1, PTrueCBlock2)
  v <- sample(1:sumJk, size = round(Perc0*sumJk), replace = F)
  PTrueC_bind[, 1][v] <- 0
  PTrueCnew <- PTrueC_bind

  XTrue <- Ttrue %*% t(PTrueCnew)
  SSXtrue <- sum(XTrue ^ 2)

  Noise <- matrix(rnorm(I*(J1+J2), mean = 0, sd = 1), I, J1+J2)
  SSNoise <- sum(Noise ^ 2)
  g <- sqrt(PropNoise*SSXtrue/(SSNoise-PropNoise*SSNoise))
  NoiseNew <- g*Noise
  #SSNoiseNew <- sum(NoiseNew ^ 2)
  Xgenerate <- XTrue + NoiseNew
  #SSXgenerate <- sum(Xgenerate ^ 2)
  #NoiseVSgenerate <- SSNoiseNew/SSXgenerate



  Data_final <- list(data = Xgenerate, T_mat = Ttrue, P_mat = PTrueCnew)
  return(Data_final)

}
```

```
##########################################################################################
######################

k <- 1
while(k <= N_dataset){

  my_data_list <- Data_generation(I, J1, J2, R, PropNoise, Perc0)

  filename <- paste("Data_", k, ".RData", sep = "")
  save(my_data_list, PropNoise, Perc0, file = filename)
  #beta_paramter <- paste("beta_", num_test, ".RData", sep = "")
  #save(beta_pre, beta1, beta2, file = beta_paramter)
  k <- k + 1

}
```

6. generating data: 4 blocks

```
set.seed(1)

Perc0 = .5   #.3 or .5
PropNoise = 0.005 # .005, .3

I <- 20
J1 <- 120
J2 <- 30
J3 <- 40
J4 <- 10
Jk <- c(J1, J2, J3, J4)
```

```
R <- 3

N_dataset <- 20  #how many datasets to generate




#########################################################################################
############################################

# A function for generating data

#########################################################################################
############################################

Data_generation <- function(I, J1, J2, J3, J4, R, PropNoise, Perc0, N_dataset){


  Jk <- c(J1, J2, J3, J4)

  sumJk <- sum(J1 + J2 + J3 + J4)


  DATA1 <- matrix(rnorm(I*J1, mean = 0, sd = 1), I, J1)

  DATA2 <- matrix(rnorm(I*J2, mean = 0, sd = 1), I, J2)

  DATA3 <- matrix(rnorm(I*J3, mean = 0, sd = 1), I, J3)

  DATA4 <- matrix(rnorm(I*J4, mean = 0, sd = 1), I, J4)

  DATA <- cbind(DATA1, DATA2, DATA3, DATA4)


  svddata <- svd(DATA, R, R)

  Ttrue <- svddata$u

  PTrueC <- as.matrix(svddata$v) %*% diag(svddata$d[1:R])   #note that only the first R eigen values are
needed.


  PTrueCBlock1 <- PTrueC[1:J1,]

  PTrueCBlock2 <- PTrueC[(J1+1):(J1+J2),]

  PTrueCBlock3 <- PTrueC[(J1+J2+1):(J1+J2+J3),]

  PTrueCBlock4 <- PTrueC[(J1+J2+J3+1):(J1+J2+J3+J4),]
```

```
PTrueCBlock1[, 3] <- 0
PTrueCBlock3[, 2] <- 0
PTrueCBlock4[, c(2,3)] <- 0

v <- sample(1:J1, size = round(Perc0*J1), replace = F)
PTrueCBlock1[, 1][v] <- 0
v <- sample(1:J1, size = round(Perc0*J1), replace = F)
PTrueCBlock1[, 2][v] <- 0

v <- sample(1:J2, size = round(Perc0*J2), replace = F)
PTrueCBlock2[, 1][v] <- 0
v <- sample(1:J2, size = round(Perc0*J2), replace = F)
PTrueCBlock2[, 2][v] <- 0
v <- sample(1:J2, size = round(Perc0*J2), replace = F)
PTrueCBlock2[, 3][v] <- 0

v <- sample(1:J3, size = round(Perc0*J3), replace = F)
PTrueCBlock3[, 1][v] <- 0
v <- sample(1:J3, size = round(Perc0*J3), replace = F)
PTrueCBlock3[, 3][v] <- 0

v <- sample(1:J4, size = round(Perc0*J4), replace = F)
PTrueCBlock4[, 1][v] <- 0

PTrueC_bind <- rbind(PTrueCBlock1, PTrueCBlock2, PTrueCBlock3, PTrueCBlock4)
PTrueCnew <- PTrueC_bind

XTrue <- Ttrue %*% t(PTrueCnew)
```

```
  SSXtrue <- sum(XTrue ^ 2)


  Noise <- matrix(rnorm(I*(J1+J2+J3+J4), mean = 0, sd = 1), I, J1+J2+J3+J4)
  SSNoise <- sum(Noise ^ 2)
  g <- sqrt(PropNoise*SSXtrue/(SSNoise-PropNoise*SSNoise))
  NoiseNew <- g*Noise
  #SSNoiseNew <- sum(NoiseNew ^ 2)
  Xgenerate <- XTrue + NoiseNew
  #SSXgenerate <- sum(Xgenerate ^ 2)
  #NoiseVSgenerate <- SSNoiseNew/SSXgenerate




  Data_final <- list(data = Xgenerate, T_mat = Ttrue, P_mat = PTrueCnew)
  return(Data_final)

}
#############################################################################################
########################


k <- 1
while(k <= N_dataset){

  my_data_list <- Data_generation(I, J1, J2, J3, J4, R, PropNoise, Perc0)

  filename <- paste("Data_", k, ".RData", sep = "")
  save(my_data_list, PropNoise, Perc0, file = filename)
  #beta_paramter <- paste("beta_", num_test, ".RData", sep = "")
  #save(beta_pre, beta1, beta2, file = beta_paramter)
```

```
  k <- k + 1

}
```

7. Empirical data

```
#######################################################
####  Illustrative applications
#######################################################
library(devtools)
install_github("ZhengguoGu/RegularizedSCA")  #load the lastest R package from Github
library(RegularizedSCA)



######### 1. analysis of the herring data  #####

#1) load the package and data, and pre-process the data
library(RegularizedSCA)
names(Herring)    #the herring data is included in the package RegularizedSCA

ChemPhy <- pre_process(Herring$Herring_ChemPhy)
Sensory <- pre_process(Herring$Herring_Sensory)
herring_data <- cbind(ChemPhy, Sensory)
num_var <- cbind(dim(ChemPhy)[2], dim(Sensory)[2])

R <- 4 # known based on previous research Gu and Van Deun 2018

Lassosequence <- seq(0.0000001, maxLGlasso(herring_data, num_var, R)$Lasso, length.out = 50)
```

```
GLassosequence <- seq(0.0000001, maxLGlasso(herring_data, num_var, R)$Glasso, length.out = 50)


#2) load function M2_BIC12andIS.R

source("M2_BIC12andIS.R")


set.seed(115)

ptm <- proc.time()

result_her_BICIS <- M2_BIC_IS(herring_data, num_var, R, LassoSequence = Lassosequence,
GLassoSequence = GLassosequence, NRSTARTS=5)


IS_index <- which(result_her_BICIS$IS == max(result_her_BICIS$IS), arr.ind = T)

Lasso_IS <- max(Lassosequence[IS_index[1]])

Glasso_IS <- max(GLassosequence[IS_index[2]])

final_her_IS <- RegularizedSCA::sparseSCA(herring_data, num_var, R, LASSO = Lasso_IS, GROUPLASSO =
Glasso_IS, MaxIter = 400, NRSTARTS = 20, method = "component")

savetime_her_IS <- proc.time() - ptm

save(final_her_IS, savetime_her_IS, file="her_IS.RData")



#4) generate a table

load("her_IS.RData")


set.seed(115)


final_her_IS <- undoShrinkage(herring_data, R,

                    final_her_IS$Pmatrix)

final_her_IS$Pmatrix

final_her_IS$Tmatrix

write.table(final_her_IS$Pmatrix, "final_herring_P.csv", sep = ",")
```

```
write.table(final_her_IS$Tmatrix, "final_herring_T.csv", sep = ",")


######## 2. analysis of metabolomics data  ########################

# 1) load data

MyData <- read.csv(file="metabolomics.csv", header=TRUE, sep=",") #cannot share on Github! Stored at the authors local computers.


meta1 <- pre_process(MyData[, 1:144])

meta2 <- pre_process(MyData[, 145:188])

metabolomics_data <- cbind(meta1, meta2)

num_var <- c(144,4)


R <- 5 # known based on previous research Gu and Van Deun 2016


Lassosequence <- seq(0.0000001, maxLGlasso(metabolomics_data , num_var, R)$Lasso, length.out = 50)

GLassosequence <- seq(0.0000001, maxLGlasso(metabolomics_data , num_var, R)$Glasso, length.out = 50)


#2) load function M2_BIC12andIS.R

source("M2_BIC12andIS.R")


set.seed(115)

ptm <- proc.time()

result_meta_BICIS <- M2_BIC_IS(metabolomics_data, num_var, R, LassoSequence = Lassosequence, GLassoSequence = GLassosequence, NRSTARTS=5)


IS_index <- which(result_meta_BICIS$IS == max(result_meta_BICIS$IS), arr.ind = T)

Lasso_IS <- max(Lassosequence[IS_index[1]])

Glasso_IS <- max(GLassosequence[IS_index[2]])
```

```
final_meta_IS <- RegularizedSCA::sparseSCA(metabolomics_data, num_var, R, LASSO = Lasso_IS, GROUPLASSO = Glasso_IS, MaxIter = 400, NRSTARTS = 20, method = "component")

savetime_meta_IS <- proc.time() - ptm

final_meta_IS$Pmatrix

save(final_meta_IS, savetime_meta_IS, file="meta_IS.RData")


set.seed(115)

final_meta_IS <- undoShrinkage(metabolomics_data, R,

                              final_meta_IS$Pmatrix)

final_meta_IS$Pmatrix


#4)  We draw a heatmap for IS

Pmat <- final_meta_IS$Pmatrix

keepname <- rownames(Pmat)

short_name <- array()  #some of the variable names are too long, we can shorten the the names (in case we want to)

for(i in 1:length(keepname)){

  short_name[i] <- substring(keepname[i], first = 1, last = 27)

}


colnames(Pmat) <- c('C1', 'C2', 'C3', 'C4', 'C5')


library(ggplot2)

names <- short_name

component <- colnames(Pmat)

PmatVec <- c(Pmat)

names <- rep(names, 5)

component <- rep(component, each = 188)
```

```
# note that part of the ggplot code below is from https://learnr.wordpress.com/2010/01/26/ggplot2-quick-heatmap-plotting/
# which is a website for drawing heatmap using ggplot2.
Pmat_dataframe <- data.frame(Loadings = PmatVec, Variables = factor(names, ordered = T, levels = short_name), Components = component)

p <- ggplot(Pmat_dataframe, aes(x = Components, y = Variables) )+
  geom_tile(aes(fill = Loadings), colour = "white") +
  scale_fill_gradient2(low="green", mid = "black", high = "red")

p + theme_grey(base_size = 15) + labs(x = "", y = "") +
  scale_x_discrete(expand = c(0, 0)) +
  scale_y_discrete(expand = c(0, 0))

########## 3. re-analysis of the parent-child relationship survey data  #########################
#1) load data
load("family_data.RData")

data<- cbind(pre_process(family_data[[1]]), pre_process(family_data[[2]]), pre_process(family_data[[3]]))
num_var <- cbind(dim(family_data[[1]])[2], dim(family_data[[2]])[2], dim(family_data[[3]])[2])
R <- 5 # known based on previous research

Lassosequence <- seq(0.0000001, maxLGlasso(data, num_var, R)$Lasso, length.out = 50)
GLassosequence <- seq(0.0000001, maxLGlasso(data, num_var, R)$Glasso, length.out = 50)

#2) load function M2_BIC12andIS.R
source("M2_BIC12andIS.R")
```

```
set.seed(115)

ptm <- proc.time()

result_fam_BICIS <- M2_BIC_IS(data, num_var, R, LassoSequence = Lassosequence, GLassoSequence =
GLassosequence, NRSTARTS=5)


IS_index <- which(result_fam_BICIS$IS == max(result_fam_BICIS$IS), arr.ind = T)

Lasso_IS <- max(Lassosequence[IS_index[1]])

Glasso_IS <- max(GLassosequence[IS_index[2]])

final_IS <- RegularizedSCA::sparseSCA(data, num_var, R, LASSO = Lasso_IS, GROUPLASSO = Glasso_IS,
MaxIter = 400, NRSTARTS = 20, method = "component")

savetime_family_IS <- proc.time() - ptm

save(final_IS, savetime_family_IS, file="family_IS.RData")


#4) Undo the shrinkage and generate a table

# In Table 2, the component loading matrix obtained from Gu and Van Deun 2018, the authors undo the
shrinkage, Hence, we undo the shrinkage here.


load("familytarget.RData")  # this is the T matrix of the Family data from Gu and Van deun 2018.


perm2 <- RegularizedSCA::TuckerCoef(family_target, final_IS$Tmatrix)$perm

final_IS_result <- final_IS$Pmatrix[, perm2]   #final P matrix for IS



set.seed(115)

final_fam_IS <- undoShrinkage(data, R = 5,

                          final_IS_result)  #position of components changed so as to be compared to the results
by RdCV

final_fam_IS$Pmatrix

write.table(final_fam_IS$Pmatrix, "final_fam.csv", sep = ",")
```

```
#!note, the final_fam_IS$Pmatrix is different from the the reported matrix in paper in terms of signs. Because regularized SCA is invariant with respect to the sign, we manually changed the signs

# so that it is easier to compared to Figure 2. The interpretation does not change as a result of change of signs.
```

8. simulation: 2 blocks

```
library(devtools)

install_github("ZhengguoGu/RegularizedSCA")

library(RegularizedSCA)

library(foreach)

library(snow)

library(doSNOW)

library(doRNG)


################# LOAD functions ########################


#please load the following functions first

#1. M1_repeatedDoubleCV.R

#2. M2_BIC12andIS.R

#3. M3_Bolasso.R

#4. M4_StabSelection.R



##################################################################################################

# StrucSCA_withIndex() estimates T and P, given the pre-defined structure of P

# This is used in M3_Bolasso.R and M4_StabSelection.R

##################################################################################################
```

```
StrucSCA_withIndex <- function (DATA, Jk, R, P_indexset, MaxIter) {

 # note that this function is needed for M3_Bolasso.R and M4_StabSelection.R
 DATA <- data.matrix(DATA)

 I_Data <- dim(DATA)[1]
 sumJk <- dim(DATA)[2]
 eps <- 10^(-12)
 if (missing(MaxIter)) {
   MaxIter <- 300
 }
 P <- matrix(stats::rnorm(sumJk * R), nrow = sumJk, ncol = R)
 P[P_indexset == 0] <- 0
 Pt <- t(P)

 residual <- sum(DATA^2)
 Lossc <- residual

 conv <- 0
 iter <- 1
 Lossvec <- array()
 while (conv == 0) {

   #### the block ########################
   A <- Pt %*% t(DATA)
   SVD_DATA <- svd(A, R, R)
   Tmat <- SVD_DATA$v %*% t(SVD_DATA$u)
   ########################################
```

```
  Lossu <- sum((DATA - Tmat %*% Pt)^2)

  P <- t(DATA) %*% Tmat
  P[P_indexset == 0] <- 0
  Pt <- t(P)

  Lossu2 <- sum((DATA - Tmat %*% Pt)^2)

  if (abs(Lossc - Lossu) < 10^(-9)) {
    Loss <- Lossu
    residual <- Lossu2
    P[abs(P) <= 2 * eps] <- 0
    conv <- 1
  }
  else if (iter > MaxIter) {
    Loss <- Lossu
    residual <- Lossu2
    P[abs(P) <= 2 * eps] <- 0
    conv <- 1
  }
  Lossvec[iter] <- Lossu
  iter <- iter + 1
  Lossc <- Lossu2
}
return_varselect <- list()
return_varselect$Pmatrix <- P
return_varselect$Tmatrix <- Tmat
return_varselect$Loss <- Loss
return_varselect$Lossvec <- Lossvec
```

```
  #return_varselect$Residual <- residual
  return(return_varselect)
}

#####################################################################################
###############
# Calculate the number of variables AND zero-loadings correctly selected
# Note: this function is about the total number of variables correctedly selected and zeros correctly
retained
#
# for the number of variables correctly selected, and the number of zero loadings correctly identified,
please see "sumarizing results.R"
#####################################################################################
##############

num_correct <- function (TargetP, EstimatedP){

  total_vnumber <- dim(TargetP)[1] * dim(TargetP)[2]

  TargetP[which(TargetP != 0)] <- 1
  sum_select <- sum(TargetP)
  sum_zero <- total_vnumber - sum_select

  EstimatedP[which(EstimatedP != 0)] <- 1

  total_correct <- sum(TargetP == EstimatedP) # this is the total number of variables correctedly selected
and zeros correctly retained

  prop_correct <- total_correct/total_vnumber
```

```
  return(prop_correct)

}


#######################################################################################
################################################

#######################################################################################
################################################


#######################################################################################
#########################

####

#### Simulations

####

#######################################################################################
#########################


N_cores <- 10 # number of cores for parallel computing


I <- 20

J1 <- 120

J2 <- 30

Jk <- c(J1, J2)

R <- 3

NRSTARTS <- 2 # #random starts

n_rep = 50    # #repetition for rdCV

n_seg = 2     # #segments for rdCV

N_boots = 50  # #repetition for BoLasso

nfolds = 5 # 5-fold CV
```

```
MaxIter = 300 # #maximum iterations

### 1. benchmark CV
set.seed(1)
n_dataset <- 1
N_dataset = 20
RESULT_BenchmarCV <- matrix(NA, N_dataset, 2)
ESTIMATED_P <- list()
ESTIMATED_T <- list()
while(n_dataset <= N_dataset){

  filename <- paste("Data_", n_dataset, ".RData", sep = "")
  load(filename)

  post_data1 <- RegularizedSCA::pre_process(my_data_list$data[, 1:J1])
  post_data2 <- RegularizedSCA::pre_process(my_data_list$data[, (J1+1):(J1+J2)])
  POST_data <- cbind(post_data1, post_data2)
  Lassosequence <- seq(0.0000001, RegularizedSCA::maxLGlasso(POST_data, Jk, R)$Lasso, length.out =
50)
  GLassosequence <- seq(0.0000001, RegularizedSCA::maxLGlasso(POST_data, Jk, R)$Glasso, length.out =
50)

  result_sim1_BM <- RegularizedSCA::cv_sparseSCA(POST_data, Jk, R, MaxIter = MaxIter, NRSTARTS,
Lassosequence, GLassosequence, nfolds, method = "component")
  tuckerresult <- RegularizedSCA::TuckerCoef(my_data_list$T_mat, result_sim1_BM$T_hat)
  RESULT_BenchmarCV[n_dataset, 1] <- tuckerresult$tucker_value
  RESULT_BenchmarCV[n_dataset, 2] <- num_correct(my_data_list$P_mat, result_sim1_BM$P_hat[,
tuckerresult$perm])

  ESTIMATED_P[[n_dataset]] <- result_sim1_BM$P_hat
```

```
  ESTIMATED_T[[n_dataset]] <- result_sim1_BM$T_hat
  n_dataset <- n_dataset + 1
}


filename <- paste("I_", I,"_J1_", J1, "_J2_", J2, "_benchmark_CV", ".RData", sep = "")
save(RESULT_BenchmarCV, ESTIMATED_P, ESTIMATED_T, file = filename)


### 2. repeated Double CV #####
n_dataset <- 1
N_dataset = 20
RESULT_rdCV <- matrix(NA, N_dataset, 2)
ESTIMATED_PrdCV <- list()
ESTIMATED_TrdCV <- list()

set.seed(1)
while(n_dataset <= N_dataset){

  filename <- paste("Data_", n_dataset, ".RData", sep = "")
  load(filename)

  post_data1 <- RegularizedSCA::pre_process(my_data_list$data[, 1:J1])
  post_data2 <- RegularizedSCA::pre_process(my_data_list$data[, (J1+1):(J1+J2)])
  POST_data <- cbind(post_data1, post_data2)

  Lassosequence <- seq(0.0000001, RegularizedSCA::maxLGlasso(POST_data, Jk, R)$Lasso, length.out =
50)
```

```
  GLassosequence <- seq(0.0000001, RegularizedSCA::maxLGlasso(POST_data, Jk, R)$Glasso, length.out = 50)

  result_sim1_RDCV <- M1_repeatedDoubleCV(POST_data,  R, Jk, N_cores, Lassosequence, GLassosequence, n_rep , n_seg, NRSTARTS, nfolds, MaxIter)

  temp_lasso <- as.data.frame(result_sim1_RDCV$Lasso)
  temp_lasso$Var1 <- sort(as.numeric(levels(temp_lasso$Var1)))
  LASSO <- max(temp_lasso[temp_lasso[,2] == max(temp_lasso[,2]),1])  #the first max ensures that the largest Lasso value is chosen, in case more than one lasso value is recommended by M1_repeatedDoubleCV
  temp_glasso <- as.data.frame(result_sim1_RDCV$GroupLasso)
  temp_glasso$Var1 <- sort(as.numeric(levels(temp_glasso$Var1)))
  GLASSO <- max(temp_glasso[temp_glasso[,2] == max(temp_glasso[,2]),1])



  final_RDCV <- RegularizedSCA::sparseSCA(POST_data, Jk, R, LASSO = LASSO, GROUPLASSO = GLASSO, MaxIter, NRSTARTS, method = "component")

  tuckerresult_RDCV <- RegularizedSCA::TuckerCoef(my_data_list$T_mat, final_RDCV$Tmatrix)
  RESULT_rdCV[n_dataset, 1] <- tuckerresult_RDCV$tucker_value
  RESULT_rdCV[n_dataset, 2] <- num_correct(my_data_list$P_mat, final_RDCV$Pmatrix[, tuckerresult_RDCV$perm])

  ESTIMATED_PrdCV[[n_dataset]] <- final_RDCV$Pmatrix
  ESTIMATED_TrdCV[[n_dataset]] <- final_RDCV$Tmatrix
  n_dataset <- n_dataset + 1

  print(n_dataset)
}
```

```
filename <- paste("I_", I,"_J1_", J1, "_J2_", J2, "_RepeatedDCV", ".RData", sep = "")
save(RESULT_rdCV, ESTIMATED_PrdCV, ESTIMATED_TrdCV, file = filename)


### 3. BIC and IS #####
n_dataset <- 1
N_dataset = 20
RESULT_BIC <- matrix(NA, N_dataset, 2)
RESULT_IS <- matrix(NA, N_dataset, 2)
ESTIMATED_Pbic <- list()
ESTIMATED_Tbic <- list()
ESTIMATED_PIS <- list()
ESTIMATED_TIS <- list()

set.seed(1)
while(n_dataset <= N_dataset){
  filename <- paste("Data_", n_dataset, ".RData", sep = "")
  load(filename)

  post_data1 <- RegularizedSCA::pre_process(my_data_list$data[, 1:J1])
  post_data2 <- RegularizedSCA::pre_process(my_data_list$data[, (J1+1):(J1+J2)])
  POST_data <- cbind(post_data1, post_data2)

  Lassosequence <- seq(0.0000001, RegularizedSCA::maxLGlasso(POST_data, Jk, R)$Lasso, length.out = 
50)
  GLassosequence <- seq(0.0000001, RegularizedSCA::maxLGlasso(POST_data, Jk, R)$Glasso, length.out = 
50)
```

```
  result_sim_BICIS <- M2_BIC_IS(POST_data, Jk, R, LassoSequence = Lassosequence, GLassoSequence = GLassosequence, NRSTARTS, MaxIter)



  Croux_index <- which(result_sim_BICIS$Croux == min(result_sim_BICIS$Croux), arr.ind = T)

  Lasso_croux <- max(Lassosequence[Croux_index[1]])  #max() is used in case multiple lasso values are chosen.

  GLasso_croux <- max(GLassosequence[Croux_index[2]])

  final_croux <- RegularizedSCA::sparseSCA(POST_data, Jk, R, LASSO = Lasso_croux, GROUPLASSO = GLasso_croux, MaxIter, NRSTARTS, method = "component")

  ESTIMATED_Pbic[[n_dataset]] <- final_croux$Pmatrix

  ESTIMATED_Tbic[[n_dataset]] <- final_croux$Tmatrix

  tuckerresult_croux <- RegularizedSCA::TuckerCoef(my_data_list$T_mat, final_croux$Tmatrix)

  RESULT_BIC[n_dataset, 1] <- tuckerresult_croux$tucker_value

  RESULT_BIC[n_dataset, 2] <- num_correct(my_data_list$P_mat, final_croux$Pmatrix[, tuckerresult_croux$perm])



  IS_index <- which(result_sim_BICIS$IS == max(result_sim_BICIS$IS), arr.ind = T)

  Lasso_IS <- max(Lassosequence[IS_index[1]])

  Glasso_IS <- max(GLassosequence[IS_index[2]])

  final_IS <- RegularizedSCA::sparseSCA(POST_data, Jk, R, LASSO = Lasso_IS, GROUPLASSO = Glasso_IS, MaxIter, NRSTARTS, method = "component")

  ESTIMATED_PIS[[n_dataset]] <- final_IS$Pmatrix

  ESTIMATED_TIS[[n_dataset]] <- final_IS$Tmatrix

  tuckerresult_IS <- RegularizedSCA::TuckerCoef(my_data_list$T_mat, final_IS$Tmatrix)

  RESULT_IS[n_dataset, 1] <- tuckerresult_IS$tucker_value

  RESULT_IS[n_dataset, 2] <- num_correct(my_data_list$P_mat, final_IS$Pmatrix[, tuckerresult_IS$perm])


  n_dataset <- n_dataset + 1
```

```
  print(n_dataset)
}

filename <- paste("I_", I,"_J1_", J1, "_J2_", J2, "_BIC_IS", ".RData", sep = "")
save(RESULT_BIC, RESULT_IS, ESTIMATED_Pbic, ESTIMATED_Tbic, ESTIMATED_PIS, ESTIMATED_TIS,  file
= filename)



### 4. Bolasso   #################
n_dataset <- 1
N_dataset = 20
RESULT_BoLasso <- matrix(NA, N_dataset, 2)
ESTIMATED_Pbolasso <- list()
ESTIMATED_Tbolasso <- list()

set.seed(1)
while(n_dataset <= N_dataset){

  filename <- paste("Data_", n_dataset, ".RData", sep = "")
  load(filename)

  post_data1 <- RegularizedSCA::pre_process(my_data_list$data[, 1:J1])
  post_data2 <- RegularizedSCA::pre_process(my_data_list$data[, (J1+1):(J1+J2)])
  POST_data <- cbind(post_data1, post_data2)

  Lassosequence <- seq(0.0000001, RegularizedSCA::maxLGlasso(POST_data, Jk, R)$Lasso, length.out =
50)
```

```
  GLassosequence <- seq(0.0000001, RegularizedSCA::maxLGlasso(POST_data, Jk, R)$Glasso, length.out = 50)

  result_sim1_Bolasso <- Bolasso_CV(POST_data, Jk, R, N_boots, LassoSequence = Lassosequence, GLassoSequence = GLassosequence, N_cores, NRSTARTS, nfolds, MaxIter)

  tuckerresult_Bolasso <- RegularizedSCA::TuckerCoef(my_data_list$T_mat, result_sim1_Bolasso$T_hat)
  RESULT_BoLasso[n_dataset, 1] <- tuckerresult_Bolasso$tucker_value
  RESULT_BoLasso[n_dataset, 2] <- num_correct(my_data_list$P_mat, result_sim1_Bolasso$P_hat[, tuckerresult_Bolasso$perm])
  ESTIMATED_Pbolasso[[n_dataset]] <- result_sim1_Bolasso$P_hat
  ESTIMATED_Tbolasso[[n_dataset]] <- result_sim1_Bolasso$T_hat

  n_dataset <- n_dataset + 1

  print(n_dataset)
}

filename <- paste("I_", I,"_J1_", J1, "_J2_", J2, "_BOLASSO", ".RData", sep = "")
save(RESULT_BoLasso, ESTIMATED_Pbolasso, ESTIMATED_Tbolasso, file = filename)


### 5. Stability selection  ############

n_dataset <- 1
N_dataset = 20
RESULT_StabS <- matrix(NA, N_dataset, 2)
ESTIMATED_PStabS <- list()
ESTIMATED_TStabS <- list()
```

```
set.seed(1)

while(n_dataset <= N_dataset){


  filename <- paste("Data_", n_dataset, ".RData", sep = "")

  load(filename)


  n_loading <- sum(my_data_list$P_mat !=0) # note! in reality we dont know the number of non-zero loadings! We just want to see if we know n_loading a priori, can the method generates good results?


  post_data1 <- RegularizedSCA::pre_process(my_data_list$data[, 1:J1])

  post_data2 <- RegularizedSCA::pre_process(my_data_list$data[, (J1+1):(J1+J2)])

  POST_data <- cbind(post_data1, post_data2)


  LassoSequence = exp(seq(from = log(0.00000001), to = log(RegularizedSCA::maxLGlasso(POST_data, Jk, R)$Lasso), length.out = 500))  #we use Lasso only

  result_sim1_StabS <- M4_StabSelection(POST_data, Jk, R, LassoSequence = LassoSequence, N_loading = n_loading, Thr = .6, NRSTARTS, N_cores, nfolds, MaxIter)


  tuckerresult_StabS <- RegularizedSCA::TuckerCoef(my_data_list$T_mat, result_sim1_StabS$T_hat)

  RESULT_StabS[n_dataset, 1] <- tuckerresult_StabS$tucker_value

  RESULT_StabS[n_dataset, 2] <- num_correct(my_data_list$P_mat, result_sim1_StabS$P_hat[, tuckerresult_StabS$perm])

  ESTIMATED_PStabS[[n_dataset]] <- result_sim1_StabS$P_hat

  ESTIMATED_TStabS[[n_dataset]] <- result_sim1_StabS$T_hat


  n_dataset <- n_dataset + 1


  print(n_dataset)

}
```

```r
filename <- paste("I_", I,"_J1_", J1, "_J2_", J2, "_Stability", ".RData", sep = "")
save(RESULT_StabS, ESTIMATED_PStabS, ESTIMATED_TStabS, file = filename)
```

9. simulation: 4 blocks

```r
library(devtools)
install_github("ZhengguoGu/RegularizedSCA")
library(RegularizedSCA)
library(foreach)
library(snow)
library(doSNOW)
library(doRNG)

################# LOAD functions #######################

#please load the following functions first
#1. M1_repeatedDoubleCV.R
#2. M2_BIC12andIS.R
#3. M3_Bolasso.R
#4. M4_StabSelection.R

##########################################################################################
#########
# StrucSCA_withIndex() estimates T and P, given the pre-defined structure of P
# This is used in M3_Bolasso.R and M4_StabSelection.R
##########################################################################################
#########
StrucSCA_withIndex <- function (DATA, Jk, R, P_indexset, MaxIter) {
```

```
# note that this function is needed for M3_Bolasso.R and M4_StabSelection.R
DATA <- data.matrix(DATA)

I_Data <- dim(DATA)[1]
sumJk <- dim(DATA)[2]
eps <- 10^(-12)
if (missing(MaxIter)) {
  MaxIter <- 300
}
P <- matrix(stats::rnorm(sumJk * R), nrow = sumJk, ncol = R)
P[P_indexset == 0] <- 0
Pt <- t(P)

residual <- sum(DATA^2)
Lossc <- residual

conv <- 0
iter <- 1
Lossvec <- array()
while (conv == 0) {

  #### the block ########################
  A <- Pt %*% t(DATA)
  SVD_DATA <- svd(A, R, R)
  Tmat <- SVD_DATA$v %*% t(SVD_DATA$u)
  ######################################

  Lossu <- sum((DATA - Tmat %*% Pt)^2)
```

```
  P <- t(DATA) %*% Tmat
  P[P_indexset == 0] <- 0
  Pt <- t(P)

  Lossu2 <- sum((DATA - Tmat %*% Pt)^2)

  if (abs(Lossc - Lossu) < 10^(-9)) {
    Loss <- Lossu
    residual <- Lossu2
    P[abs(P) <= 2 * eps] <- 0
    conv <- 1
  }
  else if (iter > MaxIter) {
    Loss <- Lossu
    residual <- Lossu2
    P[abs(P) <= 2 * eps] <- 0
    conv <- 1
  }
  Lossvec[iter] <- Lossu
  iter <- iter + 1
  Lossc <- Lossu2
}
return_varselect <- list()
return_varselect$Pmatrix <- P
return_varselect$Tmatrix <- Tmat
return_varselect$Loss <- Loss
return_varselect$Lossvec <- Lossvec
#return_varselect$Residual <- residual
```

```
  return(return_varselect)

}


###########################################################################################
###############

# Calculate the number of variables AND zero-loadings correctly selected

# Note: this function is about the total number of variables correctedly selected and zeros correctly
retained

#

# for the number of variables correctly selected, and the number of zero loadings correctly identified,
please see "sumarizing results.R"

###########################################################################################
##############


num_correct <- function (TargetP, EstimatedP){


  total_vnumber <- dim(TargetP)[1] * dim(TargetP)[2]


  TargetP[which(TargetP != 0)] <- 1

  sum_select <- sum(TargetP)

  sum_zero <- total_vnumber - sum_select


  EstimatedP[which(EstimatedP != 0)] <- 1


  total_correct <- sum(TargetP == EstimatedP) # this is the total number of variables correctedly selected
and zeros correctly retained


  prop_correct <- total_correct/total_vnumber


  return(prop_correct)
```

```
}


########################################################################################
#################################################

########################################################################################
#################################################


########################################################################################
##########################
####
#### Simulations
####
########################################################################################
##########################


N_cores <- 6 # number of cores for parallel computing (for 4blocks, I used 6 cores on blade server)


I <- 20
J1 <- 120
J2 <- 30
J3 <- 40
J4 <- 10
Jk <- c(J1, J2, J3, J4)
R <- 3
NRSTARTS <- 2 # #random starts
n_rep = 50    # #repetition for rdCV
n_seg = 2     # #segments for rdCV
N_boots = 50  # #repetition for BoLasso
```

```
nfolds = 5 # 5-fold CV

MaxIter = 300 # #maximum iterations


### 1. benchmark CV

set.seed(1)

n_dataset <- 1

N_dataset = 20

RESULT_BenchmarCV <- matrix(NA, N_dataset, 2)

ESTIMATED_P <- list()

ESTIMATED_T <- list()

while(n_dataset <= N_dataset){


  filename <- paste("Data_", n_dataset, ".RData", sep = "")

  load(filename)


  post_data1 <- RegularizedSCA::pre_process(my_data_list$data[, 1:J1])

  post_data2 <- RegularizedSCA::pre_process(my_data_list$data[, (J1+1):(J1+J2)])

  post_data3 <- RegularizedSCA::pre_process(my_data_list$data[, (J1+J2+1):(J1+J2+J3)])

  post_data4 <- RegularizedSCA::pre_process(my_data_list$data[, (J1+J2+J3+1):(J1+J2+J3+J4)])

  POST_data <- cbind(post_data1, post_data2, post_data3, post_data4)

  Lassosequence <- seq(0.0000001, RegularizedSCA::maxLGlasso(POST_data, Jk, R)$Lasso, length.out =
50)

  GLassosequence <- seq(0.0000001, RegularizedSCA::maxLGlasso(POST_data, Jk, R)$Glasso, length.out =
50)


  result_sim1_BM <- RegularizedSCA::cv_sparseSCA(POST_data, Jk, R, MaxIter = MaxIter, NRSTARTS,
Lassosequence, GLassosequence, nfolds, method = "component")

  tuckerresult <- RegularizedSCA::TuckerCoef(my_data_list$T_mat, result_sim1_BM$T_hat)

  RESULT_BenchmarCV[n_dataset, 1] <- tuckerresult$tucker_value
```

```
  RESULT_BenchmarCV[n_dataset, 2] <- num_correct(my_data_list$P_mat, result_sim1_BM$P_hat[,
tuckerresult$perm])

  ESTIMATED_P[[n_dataset]] <- result_sim1_BM$P_hat
  ESTIMATED_T[[n_dataset]] <- result_sim1_BM$T_hat
  n_dataset <- n_dataset + 1
}


filename <- paste("1_benchmark_CV", ".RData", sep = "")
save(RESULT_BenchmarCV, ESTIMATED_P, ESTIMATED_T, file = filename)


### 2. repeated Double CV #####
n_dataset <- 1
N_dataset = 20
RESULT_rdCV <- matrix(NA, N_dataset, 2)
ESTIMATED_PrdCV <- list()
ESTIMATED_TrdCV <- list()

set.seed(1)
while(n_dataset <= N_dataset){

  filename <- paste("Data_", n_dataset, ".RData", sep = "")
  load(filename)

  post_data1 <- RegularizedSCA::pre_process(my_data_list$data[, 1:J1])
  post_data2 <- RegularizedSCA::pre_process(my_data_list$data[, (J1+1):(J1+J2)])
  post_data3 <- RegularizedSCA::pre_process(my_data_list$data[, (J1+J2+1):(J1+J2+J3)])
```

```
  post_data4 <- RegularizedSCA::pre_process(my_data_list$data[, (J1+J2+J3+1):(J1+J2+J3+J4)])
  POST_data <- cbind(post_data1, post_data2, post_data3, post_data4)


  Lassosequence <- seq(0.0000001, RegularizedSCA::maxLGlasso(POST_data, Jk, R)$Lasso, length.out = 50)
  GLassosequence <- seq(0.0000001, RegularizedSCA::maxLGlasso(POST_data, Jk, R)$Glasso, length.out = 50)


  result_sim1_RDCV <- M1_repeatedDoubleCV(POST_data,  R, Jk, N_cores, Lassosequence, GLassosequence, n_rep , n_seg, NRSTARTS, nfolds, MaxIter)


  temp_lasso <- as.data.frame(result_sim1_RDCV$Lasso)
  temp_lasso$Var1 <- sort(as.numeric(levels(temp_lasso$Var1)))
  LASSO <- max(temp_lasso[temp_lasso[,2] == max(temp_lasso[,2]),1])  #the first max ensures that the largest Lasso value is chosen, in case more than one lasso value is recommended by M1_repeatedDoubleCV
  temp_glasso <- as.data.frame(result_sim1_RDCV$GroupLasso)
  temp_glasso$Var1 <- sort(as.numeric(levels(temp_glasso$Var1)))
  GLASSO <- max(temp_glasso[temp_glasso[,2] == max(temp_glasso[,2]),1])


  final_RDCV <- RegularizedSCA::sparseSCA(POST_data, Jk, R, LASSO = LASSO, GROUPLASSO = GLASSO, MaxIter, NRSTARTS, method = "component")


  tuckerresult_RDCV <- RegularizedSCA::TuckerCoef(my_data_list$T_mat, final_RDCV$Tmatrix)
  RESULT_rdCV[n_dataset, 1] <- tuckerresult_RDCV$tucker_value
  RESULT_rdCV[n_dataset, 2] <- num_correct(my_data_list$P_mat, final_RDCV$Pmatrix[, tuckerresult_RDCV$perm])


  ESTIMATED_PrdCV[[n_dataset]] <- final_RDCV$Pmatrix
  ESTIMATED_TrdCV[[n_dataset]] <- final_RDCV$Tmatrix
```

```
  n_dataset <- n_dataset + 1

  print(n_dataset)
}

filename <- paste("2_RepeatedDCV", ".RData", sep = "")
save(RESULT_rdCV, ESTIMATED_PrdCV, ESTIMATED_TrdCV, file = filename)



### 3. BIC and IS #####
n_dataset <- 1
N_dataset = 20
RESULT_BIC <- matrix(NA, N_dataset, 2)
RESULT_IS <- matrix(NA, N_dataset, 2)
ESTIMATED_Pbic <- list()
ESTIMATED_Tbic <- list()
ESTIMATED_PIS <- list()
ESTIMATED_TIS <- list()

set.seed(1)
while(n_dataset <= N_dataset){
  filename <- paste("Data_", n_dataset, ".RData", sep = "")
  load(filename)

  post_data1 <- RegularizedSCA::pre_process(my_data_list$data[, 1:J1])
  post_data2 <- RegularizedSCA::pre_process(my_data_list$data[, (J1+1):(J1+J2)])
  post_data3 <- RegularizedSCA::pre_process(my_data_list$data[, (J1+J2+1):(J1+J2+J3)])
  post_data4 <- RegularizedSCA::pre_process(my_data_list$data[, (J1+J2+J3+1):(J1+J2+J3+J4)])
```

```
  POST_data <- cbind(post_data1, post_data2, post_data3, post_data4)

  Lassosequence <- seq(0.0000001, RegularizedSCA::maxLGlasso(POST_data, Jk, R)$Lasso, length.out =
50)
  GLassosequence <- seq(0.0000001, RegularizedSCA::maxLGlasso(POST_data, Jk, R)$Glasso, length.out =
50)

  result_sim_BICIS <- M2_BIC_IS(POST_data, Jk, R, LassoSequence = Lassosequence, GLassoSequence =
GLassosequence, NRSTARTS, MaxIter)

  Croux_index <- which(result_sim_BICIS$Croux == min(result_sim_BICIS$Croux), arr.ind = T)
  Lasso_croux <- max(Lassosequence[Croux_index[1]])  #max() is used in case multiple lasso values are
chosen.
  GLasso_croux <- max(GLassosequence[Croux_index[2]])
  final_croux <- RegularizedSCA::sparseSCA(POST_data, Jk, R, LASSO = Lasso_croux, GROUPLASSO =
GLasso_croux, MaxIter, NRSTARTS, method = "component")
  ESTIMATED_Pbic[[n_dataset]] <- final_croux$Pmatrix
  ESTIMATED_Tbic[[n_dataset]] <- final_croux$Tmatrix
  tuckerresult_croux <- RegularizedSCA::TuckerCoef(my_data_list$T_mat, final_croux$Tmatrix)
  RESULT_BIC[n_dataset, 1] <- tuckerresult_croux$tucker_value
  RESULT_BIC[n_dataset, 2] <- num_correct(my_data_list$P_mat, final_croux$Pmatrix[,
tuckerresult_croux$perm])

  IS_index <- which(result_sim_BICIS$IS == max(result_sim_BICIS$IS), arr.ind = T)
  Lasso_IS <- max(Lassosequence[IS_index[1]])
  Glasso_IS <- max(GLassosequence[IS_index[2]])
  final_IS <- RegularizedSCA::sparseSCA(POST_data, Jk, R, LASSO = Lasso_IS, GROUPLASSO = Glasso_IS,
MaxIter, NRSTARTS, method = "component")
  ESTIMATED_PIS[[n_dataset]] <- final_IS$Pmatrix
```

```
  ESTIMATED_TIS[[n_dataset]] <- final_IS$Tmatrix
  tuckerresult_IS <- RegularizedSCA::TuckerCoef(my_data_list$T_mat, final_IS$Tmatrix)
  RESULT_IS[n_dataset, 1] <- tuckerresult_IS$tucker_value
  RESULT_IS[n_dataset, 2] <- num_correct(my_data_list$P_mat, final_IS$Pmatrix[,
tuckerresult_IS$perm])

  n_dataset <- n_dataset + 1

  print(n_dataset)
}

filename <- paste("3_BIC_IS", ".RData", sep = "")
save(RESULT_BIC, RESULT_IS, ESTIMATED_Pbic, ESTIMATED_Tbic, ESTIMATED_PIS, ESTIMATED_TIS,  file
= filename)




### 4. Bolasso   ##################
n_dataset <- 1
N_dataset = 20
RESULT_BoLasso <- matrix(NA, N_dataset, 2)
ESTIMATED_Pbolasso <- list()
ESTIMATED_Tbolasso <- list()

set.seed(1)
while(n_dataset <= N_dataset){

  filename <- paste("Data_", n_dataset, ".RData", sep = "")
  load(filename)
```

```
  post_data1 <- RegularizedSCA::pre_process(my_data_list$data[, 1:J1])

  post_data2 <- RegularizedSCA::pre_process(my_data_list$data[, (J1+1):(J1+J2)])

  post_data3 <- RegularizedSCA::pre_process(my_data_list$data[, (J1+J2+1):(J1+J2+J3)])

  post_data4 <- RegularizedSCA::pre_process(my_data_list$data[, (J1+J2+J3+1):(J1+J2+J3+J4)])

  POST_data <- cbind(post_data1, post_data2, post_data3, post_data4)


  Lassosequence <- seq(0.0000001, RegularizedSCA::maxLGlasso(POST_data, Jk, R)$Lasso, length.out =
50)

  GLassosequence <- seq(0.0000001, RegularizedSCA::maxLGlasso(POST_data, Jk, R)$Glasso, length.out =
50)


  result_sim1_Bolasso <- Bolasso_CV(POST_data, Jk, R, N_boots, LassoSequence = Lassosequence,
GLassoSequence = GLassosequence, N_cores, NRSTARTS, nfolds, MaxIter)


  tuckerresult_Bolasso <- RegularizedSCA::TuckerCoef(my_data_list$T_mat, result_sim1_Bolasso$T_hat)

  RESULT_BoLasso[n_dataset, 1] <- tuckerresult_Bolasso$tucker_value

  RESULT_BoLasso[n_dataset, 2] <- num_correct(my_data_list$P_mat, result_sim1_Bolasso$P_hat[,
tuckerresult_Bolasso$perm])

  ESTIMATED_Pbolasso[[n_dataset]] <- result_sim1_Bolasso$P_hat

  ESTIMATED_Tbolasso[[n_dataset]] <- result_sim1_Bolasso$T_hat


  n_dataset <- n_dataset + 1


  print(n_dataset)

}


filename <- paste("4_BOLASSO", ".RData", sep = "")

save(RESULT_BoLasso, ESTIMATED_Pbolasso, ESTIMATED_Tbolasso, file = filename)
```

```
### 5. Stability selection  #############

n_dataset <- 1
N_dataset = 20
RESULT_StabS <- matrix(NA, N_dataset, 2)
ESTIMATED_PStabS <- list()
ESTIMATED_TStabS <- list()

set.seed(1)
while(n_dataset <= N_dataset){

  filename <- paste("Data_", n_dataset, ".RData", sep = "")
  load(filename)

  n_loading <- sum(my_data_list$P_mat !=0) # note! in reality we dont know the number of non-zero loadings! We just want to see if we know n_loading a priori, can the method generates good results?

  post_data1 <- RegularizedSCA::pre_process(my_data_list$data[, 1:J1])
  post_data2 <- RegularizedSCA::pre_process(my_data_list$data[, (J1+1):(J1+J2)])
  post_data3 <- RegularizedSCA::pre_process(my_data_list$data[, (J1+J2+1):(J1+J2+J3)])
  post_data4 <- RegularizedSCA::pre_process(my_data_list$data[, (J1+J2+J3+1):(J1+J2+J3+J4)])
  POST_data <- cbind(post_data1, post_data2, post_data3, post_data4)

  LassoSequence = exp(seq(from = log(0.00000001), to = log(RegularizedSCA::maxLGlasso(POST_data, Jk, R)$Lasso), length.out = 500))  #we use Lasso only
  result_sim1_StabS <- M4_StabSelection(POST_data, Jk, R, LassoSequence = LassoSequence, N_loading = n_loading, Thr = .6, NRSTARTS, N_cores, nfolds, MaxIter)

  tuckerresult_StabS <- RegularizedSCA::TuckerCoef(my_data_list$T_mat, result_sim1_StabS$T_hat)
```

```
  RESULT_StabS[n_dataset, 1] <- tuckerresult_StabS$tucker_value
  RESULT_StabS[n_dataset, 2] <- num_correct(my_data_list$P_mat, result_sim1_StabS$P_hat[,
tuckerresult_StabS$perm])
  ESTIMATED_PStabS[[n_dataset]] <- result_sim1_StabS$P_hat
  ESTIMATED_TStabS[[n_dataset]] <- result_sim1_StabS$T_hat

  n_dataset <- n_dataset + 1

  print(n_dataset)
}

filename <- paste("5_Stability", ".RData", sep = "")
save(RESULT_StabS, ESTIMATED_PStabS, ESTIMATED_TStabS, file = filename)
```

10. summarizing results (plots etc): 2 blocks

```
###################################################################################
###################   sumarizing results of the simulation study    ############
###################             (for revision)                 ############
###################################################################################

library(ggplot2)
library(reshape2)
library(gridExtra)

################ PART 1a: Boxplots (2 data blocks), variable correctly selected and zeros correctly
identified ################################

# I. summurizing data;
```

```
# Sim_1 0.5% noise and 30% zero ######
#(note: load data by hand)
tucker_result_Sim1 <- cbind(RESULT_BenchmarCV[,1],
                    RESULT_rdCV[, 1],
                    RESULT_BIC[,1],
                    RESULT_IS[,1],
                    RESULT_BoLasso[,1],
                    RESULT_StabS[,1],
                    "0.5% noise, 30% zeros")
colnames(tucker_result_Sim1) <- c("CV", "RdCV", "BIC", "IS", "BoLasso", "Stab. selection", "condition")

PL_Sim1 <- cbind(RESULT_BenchmarCV[,2],
          RESULT_rdCV[, 2],
          RESULT_BIC[,2],
          RESULT_IS[,2],
          RESULT_BoLasso[,2],
          RESULT_StabS[,2],
          "0.5% noise, 30% zeros")
colnames(PL_Sim1) <- c("CV", "RdCV", "BIC", "IS", "BoLasso", "Stab. selection", "condition")
save(file = "sim1.RData", tucker_result_Sim1, PL_Sim1)
#######

# Sim_2 0.5% noise and 50% zero ####################
#(note: load data by hand)
tucker_result_Sim2 <- cbind(RESULT_BenchmarCV[,1],
                        RESULT_rdCV[, 1],
                        RESULT_BIC[,1],
                        RESULT_IS[,1],
```

```
                    RESULT_BoLasso[,1],
                    RESULT_StabS[,1],
                    "0.5% noise, 50% zeros")
colnames(tucker_result_Sim2) <- c("CV", "RdCV", "BIC", "IS", "BoLasso", "Stab. selection", "condition")

PL_Sim2 <- cbind(RESULT_BenchmarCV[,2],
            RESULT_rdCV[, 2],
            RESULT_BIC[,2],
            RESULT_IS[,2],
            RESULT_BoLasso[,2],
            RESULT_StabS[,2],
            "0.5% noise, 50% zeros")
colnames(PL_Sim2) <- c("CV", "RdCV", "BIC", "IS", "BoLasso", "Stab. selection", "condition")
save(file = "sim2.RData", tucker_result_Sim2, PL_Sim2)
###########

# Sim_3 30% noise and 30% zero ####################
#(note: load data by hand)
tucker_result_Sim3 <- cbind(RESULT_BenchmarCV[,1],
                    RESULT_rdCV[, 1],
                    RESULT_BIC[,1],
                    RESULT_IS[,1],
                    RESULT_BoLasso[,1],
                    RESULT_StabS[,1],
                    "30% noise, 30% zeros")
colnames(tucker_result_Sim3) <- c("CV", "RdCV", "BIC", "IS", "BoLasso", "Stab. selection", "condition")

PL_Sim3 <- cbind(RESULT_BenchmarCV[,2],
            RESULT_rdCV[, 2],
```

```
              RESULT_BIC[,2],
              RESULT_IS[,2],
              RESULT_BoLasso[,2],
              RESULT_StabS[,2],
              "30% noise, 30% zeros")
colnames(PL_Sim3) <- c("CV", "RdCV", "BIC", "IS", "BoLasso", "Stab. selection", "condition")
save(file = "sim3.RData", tucker_result_Sim3, PL_Sim3)
###########

# Sim_4 30% noise and 50% zero ####################
#(note: load data by hand)
tucker_result_Sim4 <- cbind(RESULT_BenchmarCV[,1],
                      RESULT_rdCV[, 1],
                      RESULT_BIC[,1],
                      RESULT_IS[,1],
                      RESULT_BoLasso[,1],
                      RESULT_StabS[,1],
                      "30% noise, 50% zeros")
colnames(tucker_result_Sim4) <- c("CV", "RdCV", "BIC", "IS", "BoLasso", "Stab. selection", "condition")

PL_Sim4 <- cbind(RESULT_BenchmarCV[,2],
              RESULT_rdCV[, 2],
              RESULT_BIC[,2],
              RESULT_IS[,2],
              RESULT_BoLasso[,2],
              RESULT_StabS[,2],
              "30% noise, 50% zeros")
colnames(PL_Sim4) <- c("CV", "RdCV", "BIC", "IS", "BoLasso", "Stab. selection", "condition")
save(file = "sim4.RData", tucker_result_Sim4, PL_Sim4)
```

```
###########

load("sim1.RData")
load("sim2.RData")
load("sim3.RData")
load("sim4.RData")

PL <- rbind(PL_Sim1, PL_Sim2, PL_Sim3, PL_Sim4)
PL_final<- data.frame(apply(PL[, 1:6], 2, as.numeric))
PL_final$condition <- PL[, 7]
colnames(PL_final)[c(5, 6)] <- c("BL", "SS")
dat_temp <- melt(PL_final,id.vars="condition", measure.vars=c("CV", "RdCV", "BIC", "IS", "BL", "SS"))


p <- ggplot(dat_temp, aes(x = variable, y = value)) +
  geom_boxplot()+
  scale_y_continuous(name = "Proportion of loadings correctedly selected", limits = c(0, 1)) +
  scale_x_discrete(name = "Variable selection methods") +
  ggtitle("I=80, J1=40, J2=10") +    #do not forget to manually change this.
  theme_bw() +
  theme(plot.title = element_text(size = 14, family = "Tahoma", face = "bold"),
        text = element_text(size = 14, family = "Tahoma"),
        axis.title = element_text(face="bold"),
        axis.text.x=element_text(size = 12))+
  facet_grid(. ~ condition)
p

Tucker_results <- rbind(tucker_result_Sim1, tucker_result_Sim2, tucker_result_Sim3,
tucker_result_Sim4)
```

```
Tucker_final<- data.frame(apply(Tucker_results[, 1:6], 2, as.numeric))

Tucker_final$condition <- Tucker_results[, 7]

colnames(Tucker_final)[c(5, 6)] <- c("BL", "SS")

dat_temp <- melt(Tucker_final,id.vars="condition", measure.vars=c("CV", "RdCV", "BIC", "IS", "BL",
"SS"))


p_tucker <- ggplot(dat_temp, aes(x = variable, y = value)) +

  geom_boxplot()+

  scale_y_continuous(name = "Tucker congruence", limits = c(0, 1)) +

  scale_x_discrete(name = "Variable selection methods") +

  ggtitle("I=80, J1=40, J2=10") +    #do not forget to manually change this.

  theme_bw() +

  theme(plot.title = element_text(size = 14, family = "Tahoma", face = "bold"),

        text = element_text(size = 14, family = "Tahoma"),

        axis.title = element_text(face="bold"),

        axis.text.x=element_text(size = 12))+

  facet_grid(. ~ condition)

p_tucker

############################################################################################
###########


################ PART 1b: boxplot (2 data blocks), seperately for variable correctly selected and for
zeros corrected identified ####################

# (authors' comment: first we have to record the number of non-zero loadings that are correctly
identified

# and also the number of zero loadings that are correctly identified.)


numNo0_correct <- function(MATa, MATb){

  num_corr <- sum((MATa != 0) & (MATb != 0))

  return(num_corr)
```

```
}
num0_correct <- function(MATa, MATb){
  num_corr <- sum((MATa == 0) & (MATb == 0))
  return(num_corr)
}

ratio_nonzero_zero <- function(file_names){

  #file_names: it starts with "I_20_J1_120_J2_30", or something like this.
  fnames <- paste(file_names, "_benchmark_CV.RData", sep = "")
  load(fnames)
  fnames <- paste(file_names, "_BIC_IS.RData", sep = "")
  load(fnames)
  fnames <- paste(file_names, "_BOLASSO.RData", sep = "")
  load(fnames)
  fnames <- paste(file_names, "_RepeatedDCV.RData", sep = "")
  load(fnames)
  fnames <- paste(file_names, "_Stability.RData", sep = "")
  load(fnames)

  ###
  n_dataset <- 1

  numNo0_true <- array()
  num0_true <- array()

  numNo0_crt_Benchmark <- array()   #number of non-zero loadings
  num0_Benchmark <- array()
  numNo0_crt_RdCV <- array()
```

```
  num0_RdCV <- array()
  numNo0_crt_BIC <- array()
  num0_BIC <- array()
  numNo0_crt_IS <- array()
  num0_IS <- array()
  numNo0_crt_Bolasso <- array()
  num0_Bolasso <- array()
  numNo0_crt_Stab <- array()
  num0_Stab <- array()


  while(n_dataset <= 20){

    filename <- paste("Data_", n_dataset, ".RData", sep = "")
    load(filename)

    numNo0_true[n_dataset] <- sum(my_data_list$P_mat !=0)
    num0_true[n_dataset] <- sum(my_data_list$P_mat ==0)

    #BenchmarkCV
    tuckerresult <- RegularizedSCA::TuckerCoef(my_data_list$T_mat, ESTIMATED_T[[n_dataset]])
    numNo0_crt_Benchmark[n_dataset]<- numNo0_correct(ESTIMATED_P[[n_dataset]][,
tuckerresult$perm], my_data_list$P_mat )
    num0_Benchmark[n_dataset] <- num0_correct(ESTIMATED_P[[n_dataset]][, tuckerresult$perm],
my_data_list$P_mat )

    #RdCV
    tuckerresult <- RegularizedSCA::TuckerCoef(my_data_list$T_mat, ESTIMATED_TrdCV[[n_dataset]])
    numNo0_crt_RdCV[n_dataset] <- numNo0_correct(ESTIMATED_PrdCV[[n_dataset]][,
tuckerresult$perm], my_data_list$P_mat )
```

```
    num0_RdCV[n_dataset]<- num0_correct(ESTIMATED_PrdCV[[n_dataset]][, tuckerresult$perm],
my_data_list$P_mat )


    #BIC

    tuckerresult <- RegularizedSCA::TuckerCoef(my_data_list$T_mat, ESTIMATED_Tbic[[n_dataset]])

    numNo0_crt_BIC[n_dataset] <- numNo0_correct(ESTIMATED_Pbic[[n_dataset]][, tuckerresult$perm],
my_data_list$P_mat )

    num0_BIC[n_dataset] <- num0_correct(ESTIMATED_Pbic[[n_dataset]][, tuckerresult$perm],
my_data_list$P_mat )


    #IS

    tuckerresult <- RegularizedSCA::TuckerCoef(my_data_list$T_mat, ESTIMATED_TIS[[n_dataset]])

    numNo0_crt_IS[n_dataset] <- numNo0_correct(ESTIMATED_PIS[[n_dataset]][, tuckerresult$perm],
my_data_list$P_mat )

    num0_IS[n_dataset] <- num0_correct(ESTIMATED_PIS[[n_dataset]][, tuckerresult$perm],
my_data_list$P_mat )


    #Bolasso

    tuckerresult <- RegularizedSCA::TuckerCoef(my_data_list$T_mat, ESTIMATED_Tbolasso[[n_dataset]])

    numNo0_crt_Bolasso[n_dataset] <- numNo0_correct(ESTIMATED_Pbolasso[[n_dataset]][,
tuckerresult$perm], my_data_list$P_mat )

    num0_Bolasso[n_dataset] <- num0_correct(ESTIMATED_Pbolasso[[n_dataset]][, tuckerresult$perm],
my_data_list$P_mat )


    #Stability Selection

    tuckerresult <- RegularizedSCA::TuckerCoef(my_data_list$T_mat, ESTIMATED_TStabS[[n_dataset]])

    numNo0_crt_Stab[n_dataset] <- numNo0_correct(ESTIMATED_PStabS[[n_dataset]][,
tuckerresult$perm], my_data_list$P_mat )

    num0_Stab[n_dataset] <- num0_correct(ESTIMATED_PStabS[[n_dataset]][, tuckerresult$perm],
my_data_list$P_mat )


    n_dataset <- n_dataset + 1
```

```
  }

  Ratio_numNo0_crt_Benchmark <- numNo0_crt_Benchmark / numNo0_true
  Ratio_num0_Benchmark <- num0_Benchmark / num0_true
  Ratio_numNo0_crt_RdCV <- numNo0_crt_RdCV / numNo0_true
  Ratio_num0_RdCV <- num0_RdCV / num0_true
  Ratio_numNo0_crt_BIC <- numNo0_crt_BIC / numNo0_true
  Ratio_num0_BIC <- num0_BIC / num0_true
  Ratio_numNo0_crt_IS <- numNo0_crt_IS / numNo0_true
  Ratio_num0_IS <- num0_IS / num0_true
  Ratio_numNo0_crt_Bolasso <- numNo0_crt_Bolasso / numNo0_true
  Ratio_num0_Bolasso <- num0_Bolasso / num0_true
  Ratio_numNo0_crt_Stab <- numNo0_crt_Stab / numNo0_true
  Ratio_num0_Stab <- num0_Stab / num0_true

  result <- cbind(Ratio_numNo0_crt_Benchmark, Ratio_numNo0_crt_RdCV, Ratio_numNo0_crt_BIC,
Ratio_numNo0_crt_IS, Ratio_numNo0_crt_Bolasso, Ratio_numNo0_crt_Stab,
                  Ratio_num0_Benchmark, Ratio_num0_RdCV, Ratio_num0_BIC, Ratio_num0_IS,
Ratio_num0_Bolasso, Ratio_num0_Stab)
  return(result)
}

### folder 2block_I20_J120_30
file_heading <- "I_20_J1_120_J2_30"
# note, change directory to the correct subfolder: for example, for "Sim_1 0.5% noise and 30% zero"
(see below), change directory to the 0_005noise_0_3zeros folder
# Sim_1 0.5% noise and 30% zero ######
result_sim1 <- ratio_nonzero_zero(file_heading)
save(result_sim1, file = "seperate_sim1.RData")
# Sim_2 0.5% noise and 50% zero ######
```

```
result_sim2 <- ratio_nonzero_zero(file_heading)
save(result_sim2, file = "seperate_sim2.RData")
# Sim_3 30% noise and 30% zero #######
result_sim3 <- ratio_nonzero_zero(file_heading)
save(result_sim3, file = "seperate_sim3.RData")
# Sim_4 30% noise and 50% zero #######
result_sim4 <- ratio_nonzero_zero(file_heading)
save(result_sim4, file = "seperate_sim4.RData")

### folder 2block_I20_J40_10
file_heading <- "I_20_J1_40_J2_10"
# Sim_1 0.5% noise and 30% zero ######
result_sim1 <- ratio_nonzero_zero(file_heading)
save(result_sim1, file = "seperate_sim1.RData")
# Sim_2 0.5% noise and 50% zero ######
result_sim2 <- ratio_nonzero_zero(file_heading)
save(result_sim2, file = "seperate_sim2.RData")
# Sim_3 30% noise and 30% zero #######
result_sim3 <- ratio_nonzero_zero(file_heading)
save(result_sim3, file = "seperate_sim3.RData")
# Sim_4 30% noise and 50% zero #######
result_sim4 <- ratio_nonzero_zero(file_heading)
save(result_sim4, file = "seperate_sim4.RData")

### folder 2block_I80_J40_10
file_heading <- "I_80_J1_40_J2_10"
# Sim_1 0.5% noise and 30% zero ######
result_sim1 <- ratio_nonzero_zero(file_heading)
save(result_sim1, file = "seperate_sim1.RData")
```

```
# Sim_2 0.5% noise and 50% zero ######
result_sim2 <- ratio_nonzero_zero(file_heading)
save(result_sim2, file = "seperate_sim2.RData")
# Sim_3 30% noise and 30% zero #######
result_sim3 <- ratio_nonzero_zero(file_heading)
save(result_sim3, file = "seperate_sim3.RData")
# Sim_4 30% noise and 50% zero #######
result_sim4 <- ratio_nonzero_zero(file_heading)
save(result_sim4, file = "seperate_sim4.RData")

###### boxplots
library(ggplot2)
library(reshape2)
library(gridExtra)

PL1_sim1 <- data.frame(result_sim1[, 1:6])  #variables correctly identified
PL1_sim1$condition <- "0.5% noise and 30% zero"
PL2_sim1 <- data.frame(result_sim1[, 7:12])  #zeros correctly identified
PL2_sim1$condition <- "0.5% noise and 30% zero"
PL1_sim2 <- data.frame(result_sim2[, 1:6])  #variables correctly identified
PL1_sim2$condition <- "0.5% noise and 50% zero"
PL2_sim2 <- data.frame(result_sim2[, 7:12])  #zeros correctly identified
PL2_sim2$condition <- "0.5% noise and 50% zero"
PL1_sim3 <- data.frame(result_sim3[, 1:6])  #variables correctly identified
PL1_sim3$condition <- "30% noise and 30% zero"
PL2_sim3 <- data.frame(result_sim3[, 7:12])  #zeros correctly identified
PL2_sim3$condition <- "30% noise and 30% zero"
PL1_sim4 <- data.frame(result_sim4[, 1:6])  #variables correctly identified
PL1_sim4$condition <- "30% noise and 50% zero"
```

```
PL2_sim4 <- data.frame(result_sim4[, 7:12])  #zeros correctly identified
PL2_sim4$condition <- "30% noise and 50% zero"


PL1 <- rbind(PL1_sim1, PL1_sim2, PL1_sim3, PL1_sim4)
colnames(PL1)[1:6] <- c("CV", "RdCV", "BIC", "IS", "BL", "SS")
dat_temp <- melt(PL1,id.vars="condition", measure.vars=c("CV", "RdCV", "BIC", "IS", "BL", "SS"))
p <- ggplot(dat_temp, aes(x = variable, y = value)) +
  geom_boxplot()+
  scale_y_continuous(name = "Proportion of non-zero loadings correctedly selected", limits = c(0, 1)) +
  scale_x_discrete(name = "Variable selection methods") +
  ggtitle("I=80, J1=40, J2=10") +    #do not forget to manually change this.
  theme_bw() +
  theme(plot.title = element_text(size = 14, family = "Tahoma", face = "bold"),
        text = element_text(size = 14, family = "Tahoma"),
        axis.title = element_text(face="bold"),
        axis.text.x=element_text(size = 12))+
  facet_grid(. ~ condition)
p


PL2 <- rbind(PL2_sim1, PL2_sim2, PL2_sim3, PL2_sim4)
colnames(PL2)[1:6] <- c("CV", "RdCV", "BIC", "IS", "BL", "SS")
dat_temp <- melt(PL2,id.vars="condition", measure.vars=c("CV", "RdCV", "BIC", "IS", "BL", "SS"))
p <- ggplot(dat_temp, aes(x = variable, y = value)) +
  geom_boxplot()+
  scale_y_continuous(name = "Proportion of zero loadings correctedly identified", limits = c(0, 1)) +
  scale_x_discrete(name = "Variable selection methods") +
  ggtitle("I=80, J1=40, J2=10") +    #do not forget to manually change this.
  theme_bw() +
  theme(plot.title = element_text(size = 14, family = "Tahoma", face = "bold"),
```

```
        text = element_text(size = 14, family = "Tahoma"),
        axis.title = element_text(face="bold"),
        axis.text.x=element_text(size = 12))+
  facet_grid(. ~ condition)
p
```

11. summarizing results 4 blocks

```
##########################################################################################
###################   sumarizing results of the simulation study    ############
###################              (for revision)                  ############
##########################################################################################

library(ggplot2)
library(reshape2)
library(gridExtra)
library(devtools)
install_github("ZhengguoGu/RegularizedSCA")
library(RegularizedSCA)

################ PART 1a: Boxplots (4 data blocks), variable correctly selected and zeros correctly identified ################################

# I. summurizing data;

# Sim_1 0.5% noise and 30% zero ######
#(note: load data by hand)
tucker_result_Sim1 <- cbind(RESULT_BenchmarCV[,1],
```

```
                RESULT_rdCV[, 1],
                RESULT_BIC[,1],
                RESULT_IS[,1],
                RESULT_BoLasso[,1],
                RESULT_StabS[,1],
                "0.5% noise, 30% zeros")
colnames(tucker_result_Sim1) <- c("CV", "RdCV", "BIC", "IS", "BoLasso", "Stab. selection", "condition")

PL_Sim1 <- cbind(RESULT_BenchmarCV[,2],
        RESULT_rdCV[, 2],
        RESULT_BIC[,2],
        RESULT_IS[,2],
        RESULT_BoLasso[,2],
        RESULT_StabS[,2],
        "0.5% noise, 30% zeros")
colnames(PL_Sim1) <- c("CV", "RdCV", "BIC", "IS", "BoLasso", "Stab. selection", "condition")
save(file = "sim1.RData", tucker_result_Sim1, PL_Sim1)
#######

# Sim_2 0.5% noise and 50% zero ####################
#(note: load data by hand)
tucker_result_Sim2 <- cbind(RESULT_BenchmarCV[,1],
                  RESULT_rdCV[, 1],
                  RESULT_BIC[,1],
                  RESULT_IS[,1],
                  RESULT_BoLasso[,1],
                  RESULT_StabS[,1],
                  "0.5% noise, 50% zeros")
colnames(tucker_result_Sim2) <- c("CV", "RdCV", "BIC", "IS", "BoLasso", "Stab. selection", "condition")
```

```
PL_Sim2 <- cbind(RESULT_BenchmarCV[,2],
          RESULT_rdCV[, 2],
          RESULT_BIC[,2],
          RESULT_IS[,2],
          RESULT_BoLasso[,2],
          RESULT_StabS[,2],
          "0.5% noise, 50% zeros")
colnames(PL_Sim2) <- c("CV", "RdCV", "BIC", "IS", "BoLasso", "Stab. selection", "condition")
save(file = "sim2.RData", tucker_result_Sim2, PL_Sim2)
###########

# Sim_3 30% noise and 30% zero ####################
#(note: load data by hand)
tucker_result_Sim3 <- cbind(RESULT_BenchmarCV[,1],
               RESULT_rdCV[, 1],
               RESULT_BIC[,1],
               RESULT_IS[,1],
               RESULT_BoLasso[,1],
               RESULT_StabS[,1],
               "30% noise, 30% zeros")
colnames(tucker_result_Sim3) <- c("CV", "RdCV", "BIC", "IS", "BoLasso", "Stab. selection", "condition")

PL_Sim3 <- cbind(RESULT_BenchmarCV[,2],
          RESULT_rdCV[, 2],
          RESULT_BIC[,2],
          RESULT_IS[,2],
          RESULT_BoLasso[,2],
          RESULT_StabS[,2],
```

```
                "30% noise, 30% zeros")
colnames(PL_Sim3) <- c("CV", "RdCV", "BIC", "IS", "BoLasso", "Stab. selection", "condition")
save(file = "sim3.RData", tucker_result_Sim3, PL_Sim3)
###########

# Sim_4 30% noise and 50% zero ####################
#(note: load data by hand)
tucker_result_Sim4 <- cbind(RESULT_BenchmarCV[,1],
                            RESULT_rdCV[, 1],
                            RESULT_BIC[,1],
                            RESULT_IS[,1],
                            RESULT_BoLasso[,1],
                            RESULT_StabS[,1],
                            "30% noise, 50% zeros")
colnames(tucker_result_Sim4) <- c("CV", "RdCV", "BIC", "IS", "BoLasso", "Stab. selection", "condition")

PL_Sim4 <- cbind(RESULT_BenchmarCV[,2],
                RESULT_rdCV[, 2],
                RESULT_BIC[,2],
                RESULT_IS[,2],
                RESULT_BoLasso[,2],
                RESULT_StabS[,2],
                "30% noise, 50% zeros")
colnames(PL_Sim4) <- c("CV", "RdCV", "BIC", "IS", "BoLasso", "Stab. selection", "condition")
save(file = "sim4.RData", tucker_result_Sim4, PL_Sim4)
###########

load("sim1.RData")
load("sim2.RData")
```

```
load("sim3.RData")
load("sim4.RData")

PL <- rbind(PL_Sim1, PL_Sim2, PL_Sim3, PL_Sim4)
PL_final<- data.frame(apply(PL[, 1:6], 2, as.numeric))
PL_final$condition <- PL[, 7]
colnames(PL_final)[c(5, 6)] <- c("BL", "SS")
dat_temp <- melt(PL_final,id.vars="condition", measure.vars=c("CV", "RdCV", "BIC", "IS", "BL", "SS"))


p <- ggplot(dat_temp, aes(x = variable, y = value)) +
  geom_boxplot()+
  scale_y_continuous(name = "Proportion of loadings correctedly selected", limits = c(0, 1)) +
  scale_x_discrete(name = "Variable selection methods (4 blocks)") +
  #ggtitle("I=80, J1=40, J2=10") +    #do not forget to manually change this.
  theme_bw() +
  theme(plot.title = element_text(size = 14, family = "Tahoma", face = "bold"),
        text = element_text(size = 14, family = "Tahoma"),
        axis.title = element_text(face="bold"),
        axis.text.x=element_text(size = 12))+
  facet_grid(. ~ condition)
p

Tucker_results <- rbind(tucker_result_Sim1, tucker_result_Sim2, tucker_result_Sim3,
tucker_result_Sim4)
Tucker_final<- data.frame(apply(Tucker_results[, 1:6], 2, as.numeric))
Tucker_final$condition <- Tucker_results[, 7]
colnames(Tucker_final)[c(5, 6)] <- c("BL", "SS")
```

```
dat_temp <- melt(Tucker_final,id.vars="condition", measure.vars=c("CV", "RdCV", "BIC", "IS", "BL",
"SS"))

p_tucker <- ggplot(dat_temp, aes(x = variable, y = value)) +
  geom_boxplot()+
  scale_y_continuous(name = "Tucker congruence", limits = c(0, 1)) +
  scale_x_discrete(name = "Variable selection methods (4 blocks)") +
  #ggtitle("I=80, J1=40, J2=10") +    #do not forget to manually change this.
  theme_bw() +
  theme(plot.title = element_text(size = 14, family = "Tahoma", face = "bold"),
        text = element_text(size = 14, family = "Tahoma"),
        axis.title = element_text(face="bold"),
        axis.text.x=element_text(size = 12))+
  facet_grid(. ~ condition)
p_tucker
########################################################################################
###########

################ PART 1b: boxplot (4 data blocks), seperately for variable correctly selected and for
zeros corrected identified ####################
# (authors' comment: first we have to record the number of non-zero loadings that are correctly
identified
# and also the number of zero loadings that are correctly identified.)

numNo0_correct <- function(MATa, MATb){
  num_corr <- sum((MATa != 0) & (MATb != 0))
  return(num_corr)
}
num0_correct <- function(MATa, MATb){
  num_corr <- sum((MATa == 0) & (MATb == 0))
```

```
  return(num_corr)
}

ratio_nonzero_zero <- function(){

  #file_names: it starts with "I_20_J1_120_J2_30", or something like this.
  fnames <- "1_benchmark_CV.RData"
  load(fnames)
  fnames <- "3_BIC_IS.RData"
  load(fnames)
  fnames <- "4_BOLASSO.RData"
  load(fnames)
  fnames <- "2_RepeatedDCV.RData"
  load(fnames)
  fnames <- "5_Stability.RData"
  load(fnames)

  ###
  n_dataset <- 1

  numNo0_true <- array()
  num0_true <- array()

  numNo0_crt_Benchmark <- array()   #number of non-zero loadings
  num0_Benchmark <- array()
  numNo0_crt_RdCV <- array()
  num0_RdCV <- array()
  numNo0_crt_BIC <- array()
  num0_BIC <- array()
```

```r
  numNo0_crt_IS <- array()
  num0_IS <- array()
  numNo0_crt_Bolasso <- array()
  num0_Bolasso <- array()
  numNo0_crt_Stab <- array()
  num0_Stab <- array()


  while(n_dataset <= 20){

    filename <- paste("Data_", n_dataset, ".RData", sep = "")
    load(filename)

    numNo0_true[n_dataset] <- sum(my_data_list$P_mat !=0)
    num0_true[n_dataset] <- sum(my_data_list$P_mat ==0)

    #BenchmarkCV
    tuckerresult <- RegularizedSCA::TuckerCoef(my_data_list$T_mat, ESTIMATED_T[[n_dataset]])
    numNo0_crt_Benchmark[n_dataset]<- numNo0_correct(ESTIMATED_P[[n_dataset]][,
tuckerresult$perm], my_data_list$P_mat )
    num0_Benchmark[n_dataset] <- num0_correct(ESTIMATED_P[[n_dataset]][, tuckerresult$perm],
my_data_list$P_mat )

    #RdCV
    tuckerresult <- RegularizedSCA::TuckerCoef(my_data_list$T_mat, ESTIMATED_TrdCV[[n_dataset]])
    numNo0_crt_RdCV[n_dataset] <- numNo0_correct(ESTIMATED_PrdCV[[n_dataset]][,
tuckerresult$perm], my_data_list$P_mat )
    num0_RdCV[n_dataset]<- num0_correct(ESTIMATED_PrdCV[[n_dataset]][, tuckerresult$perm],
my_data_list$P_mat )
```

```
    #BIC

    tuckerresult <- RegularizedSCA::TuckerCoef(my_data_list$T_mat, ESTIMATED_Tbic[[n_dataset]])

    numNo0_crt_BIC[n_dataset] <- numNo0_correct(ESTIMATED_Pbic[[n_dataset]][, tuckerresult$perm],
my_data_list$P_mat )

    num0_BIC[n_dataset] <- num0_correct(ESTIMATED_Pbic[[n_dataset]][, tuckerresult$perm],
my_data_list$P_mat )


    #IS

    tuckerresult <- RegularizedSCA::TuckerCoef(my_data_list$T_mat, ESTIMATED_TIS[[n_dataset]])

    numNo0_crt_IS[n_dataset] <- numNo0_correct(ESTIMATED_PIS[[n_dataset]][, tuckerresult$perm],
my_data_list$P_mat )

    num0_IS[n_dataset] <- num0_correct(ESTIMATED_PIS[[n_dataset]][, tuckerresult$perm],
my_data_list$P_mat )


    #Bolasso

    tuckerresult <- RegularizedSCA::TuckerCoef(my_data_list$T_mat, ESTIMATED_Tbolasso[[n_dataset]])

    numNo0_crt_Bolasso[n_dataset] <- numNo0_correct(ESTIMATED_Pbolasso[[n_dataset]][,
tuckerresult$perm], my_data_list$P_mat )

    num0_Bolasso[n_dataset] <- num0_correct(ESTIMATED_Pbolasso[[n_dataset]][, tuckerresult$perm],
my_data_list$P_mat )


    #Stability Selection

    tuckerresult <- RegularizedSCA::TuckerCoef(my_data_list$T_mat, ESTIMATED_TStabS[[n_dataset]])

    numNo0_crt_Stab[n_dataset] <- numNo0_correct(ESTIMATED_PStabS[[n_dataset]][,
tuckerresult$perm], my_data_list$P_mat )

    num0_Stab[n_dataset] <- num0_correct(ESTIMATED_PStabS[[n_dataset]][, tuckerresult$perm],
my_data_list$P_mat )


    n_dataset <- n_dataset + 1

  }


  Ratio_numNo0_crt_Benchmark <- numNo0_crt_Benchmark / numNo0_true
```

```
  Ratio_num0_Benchmark <- num0_Benchmark / num0_true
  Ratio_numNo0_crt_RdCV <- numNo0_crt_RdCV / numNo0_true
  Ratio_num0_RdCV <- num0_RdCV / num0_true
  Ratio_numNo0_crt_BIC <- numNo0_crt_BIC / numNo0_true
  Ratio_num0_BIC <- num0_BIC / num0_true
  Ratio_numNo0_crt_IS <- numNo0_crt_IS / numNo0_true
  Ratio_num0_IS <- num0_IS / num0_true
  Ratio_numNo0_crt_Bolasso <- numNo0_crt_Bolasso / numNo0_true
  Ratio_num0_Bolasso <- num0_Bolasso / num0_true
  Ratio_numNo0_crt_Stab <- numNo0_crt_Stab / numNo0_true
  Ratio_num0_Stab <- num0_Stab / num0_true

  result <- cbind(Ratio_numNo0_crt_Benchmark, Ratio_numNo0_crt_RdCV, Ratio_numNo0_crt_BIC,
Ratio_numNo0_crt_IS, Ratio_numNo0_crt_Bolasso, Ratio_numNo0_crt_Stab,
                  Ratio_num0_Benchmark, Ratio_num0_RdCV, Ratio_num0_BIC, Ratio_num0_IS,
Ratio_num0_Bolasso, Ratio_num0_Stab)
  return(result)
}

### folder 2block_I20_J120_30

# note, change directory to the correct subfolder
# Sim_1 0.5% noise and 30% zero ######
result_sim1 <- ratio_nonzero_zero()
save(result_sim1, file = "seperate_sim1.RData")
# Sim_2 0.5% noise and 50% zero ######
result_sim2 <- ratio_nonzero_zero()
save(result_sim2, file = "seperate_sim2.RData")
# Sim_3 30% noise and 30% zero #######
```

```
result_sim3 <- ratio_nonzero_zero()

save(result_sim3, file = "seperate_sim3.RData")

# Sim_4 30% noise and 50% zero #######

result_sim4 <- ratio_nonzero_zero()

save(result_sim4, file = "seperate_sim4.RData")



###### boxplots

library(ggplot2)

library(reshape2)

library(gridExtra)


PL1_sim1 <- data.frame(result_sim1[, 1:6])  #variables correctly identified

PL1_sim1$condition <- "0.5% noise and 30% zero"

PL2_sim1 <- data.frame(result_sim1[, 7:12])  #zeros correctly identified

PL2_sim1$condition <- "0.5% noise and 30% zero"

PL1_sim2 <- data.frame(result_sim2[, 1:6])  #variables correctly identified

PL1_sim2$condition <- "0.5% noise and 50% zero"

PL2_sim2 <- data.frame(result_sim2[, 7:12])  #zeros correctly identified

PL2_sim2$condition <- "0.5% noise and 50% zero"

PL1_sim3 <- data.frame(result_sim3[, 1:6])  #variables correctly identified

PL1_sim3$condition <- "30% noise and 30% zero"

PL2_sim3 <- data.frame(result_sim3[, 7:12])  #zeros correctly identified

PL2_sim3$condition <- "30% noise and 30% zero"

PL1_sim4 <- data.frame(result_sim4[, 1:6])  #variables correctly identified

PL1_sim4$condition <- "30% noise and 50% zero"

PL2_sim4 <- data.frame(result_sim4[, 7:12])  #zeros correctly identified

PL2_sim4$condition <- "30% noise and 50% zero"
```

```
PL1 <- rbind(PL1_sim1, PL1_sim2, PL1_sim3, PL1_sim4)
colnames(PL1)[1:6] <- c("CV", "RdCV", "BIC", "IS", "BL", "SS")
dat_temp <- melt(PL1,id.vars="condition", measure.vars=c("CV", "RdCV", "BIC", "IS", "BL", "SS"))
p <- ggplot(dat_temp, aes(x = variable, y = value)) +
  geom_boxplot()+
  scale_y_continuous(name = "Proportion of non-zero loadings correctedly selected", limits = c(0, 1)) +
  scale_x_discrete(name = "Variable selection methods (4 blocks)") +
  #ggtitle("I=80, J1=40, J2=10") +    #do not forget to manually change this.
  theme_bw() +
  theme(plot.title = element_text(size = 14, family = "Tahoma", face = "bold"),
        text = element_text(size = 14, family = "Tahoma"),
        axis.title = element_text(face="bold"),
        axis.text.x=element_text(size = 12))+
  facet_grid(. ~ condition)
p

PL2 <- rbind(PL2_sim1, PL2_sim2, PL2_sim3, PL2_sim4)
colnames(PL2)[1:6] <- c("CV", "RdCV", "BIC", "IS", "BL", "SS")
dat_temp <- melt(PL2,id.vars="condition", measure.vars=c("CV", "RdCV", "BIC", "IS", "BL", "SS"))
p <- ggplot(dat_temp, aes(x = variable, y = value)) +
  geom_boxplot()+
  scale_y_continuous(name = "Proportion of zero loadings correctedly identified", limits = c(0, 1)) +
  scale_x_discrete(name = "Variable selection methods (4 blocks)") +
  #ggtitle("I=80, J1=40, J2=10") +    #do not forget to manually change this.
  theme_bw() +
  theme(plot.title = element_text(size = 14, family = "Tahoma", face = "bold"),
        text = element_text(size = 14, family = "Tahoma"),
        axis.title = element_text(face="bold"),
        axis.text.x=element_text(size = 12))+
```

```
  facet_grid(. ~ condition)
p
```
